# Deciphering Cowpea Resistance to Potyvirus: Assessment of *eIF4E* Gene Mutations and Their Impact on the eIF4E-VPg Protein Interaction

**DOI:** 10.3390/v17081050

**Published:** 2025-07-28

**Authors:** Fernanda Alves de Andrade, Madson Allan de Luna-Aragão, José Diogo Cavalcanti Ferreira, Fernanda Freitas Souza, Ana Carolina da Rocha Oliveira, Antônio Félix da Costa, Francisco José Lima Aragão, Carlos André dos Santos-Silva, Ana Maria Benko-Iseppon, Valesca Pandolfi

**Affiliations:** 1 Centro de Biociências, Universidade Federal de Pernambuco (UFPE), Recife 50670-901, Brazil; fernanda.aandrade@ufpe.br (F.A.d.A.); fernanda.freitass@ufpe.br (F.F.S.); carolina.roliveira@ufpe.br (A.C.d.R.O.); 2 Instituto de Ciências Biológicas, Universidade Federal de Minas Gerais (UFMG), Belo Horizonte 31270-901, Brazil; madsondeluna@gmail.com; 3 Instituto Federal de Educação, Ciência e Tecnologia de Pernambuco (IFPE), Abreu e Lima 53515-120, Brazil; jdiogocavalcantif@yahoo.com.br; 4 Instituto Agronômico de Pernambuco (IPA), Recife 50761-000, Brazil; afelixc.ipa@gmail.com; 5 Embrapa Recursos Genéticos e Biotecnologia, PqEB W5 Norte, Brasília 70770-900, Brazil; francisco.aragao@embrapa.br; 6 Centro Universitário CESMAC, Maceió 57051-160, Brazil; carlos.biomedicina@gmail.com

**Keywords:** *Vigna unguiculata*, translation initiation factor 4E, CABMV, recessive resistance

## Abstract

Cowpea (*Vigna unguiculata*) is a crop of significant socioeconomic importance, particularly in the semi-arid regions of Africa and America. However, its productivity has been adversely affected by viral diseases, including the *cowpea aphid-borne mosaic virus* (CABMV), a single-stranded RNA virus. It is known that the VPg protein interacts with the host’s translation initiation factor (eIF4E), promoting viral replication. This study aimed to investigate the relationship between mutations in the cowpea *eIF4E* gene and resistance to CABMV. Twenty-seven cultivars were screened by PCR and bioassays for presence/absence of mutations associated with resistance or susceptibility to Potyviruses. Of the cultivars with mutations previously associated with susceptibility, 88.24% exhibited viral symptoms, while 62.5% associated with resistance remained asymptomatic. The in silico analyses revealed that non-synonymous mutations (Pro68Arg, Gly109Arg) alter the structure of the eIF4E protein, reducing its affinity to VPg. Molecular dynamics simulations also pointed to an enhanced structural stability of eIF4E in resistant cultivars and reinforced, for the first time, key mutations and the functional role of the *eIF4E* gene in resistance to CABMV in cowpea. Our results offer valuable insights for virus disease management and for genetic improvement programs for this important crop.

## 1. Introduction

Cowpea (*Vigna unguiculata* (L.) Walp) is a multifunctional legume known for its ability to tolerate poor soils and hot environments [1]. The cultivation of this legume contributes to soil quality due to its ability to fix nitrogen through symbiosis with rhizobia bacteria [2]. In addition to its high adaptability, cowpea stands out for its remarkable nutritional value, with about 32% protein and 62% carbohydrates, besides a high content of essential amino acids, vitamins, and minerals [3,4]. It is a crop of socioeconomic interest, prevalent in semi-arid regions around the world, mainly in countries of America and Africa [5].

Although this crop exhibits adaptability to unfavorable environments, its productivity has been negatively impacted in various regions worldwide due to the incidence of phytopathogens and pests, including plant viruses [6]. Among the major plant viruses affecting cowpea, two are particularly noteworthy: *cowpea severe mosaic virus* (CPSMV), from the Secoviridae family (genus Comovirus), and *cowpea aphid-borne mosaic virus* (CABMV), a member of the Potyviridae family (genus Potyvirus), which is transmitted by aphids [7,8]. Of these, CABMV is considered one of the most widespread and damaging viruses, directly impacting production and, consequently, having a major economic impact on the agro-industrial sector [9]. Viruses of the Potyviridae family exhibit a monopartite single-stranded positive-sense RNA (+ssRNA) genome that encodes ten viral proteins. The 5’ end of the viral RNA is covalently linked to the viral protein genome-linked (VPg), while the 3’ end has a polyadenylated tail (Poly-A) [10]. VPg is part of the group of proteins present in viruses of the Potyviridae family [11]. In Potyvirus, VPg is correlated with the hijacking and utilization of the plant host’s translation machinery through association with translation initiation factor 4E (eIF4E), which is used to complete the viral replication cycle [12]. The eIF4E-VPg interaction occurs due to the physicochemical characteristics of VPg, which mimics and competes with the 7-methylguanosine cap (m7GpppN), found at the 5’ end of the host’s messenger RNA (mRNA), by binding to the cap-binding domain of eIF4E [13]. A study on the VPg of *potato virus Y* (PVY) revealed, through nuclear magnetic resonance (NMR) and molecular docking, that the VPg region binding to eIF4E has a negative surface charge. In contrast, the binding domain of eIF4E has a predominantly positive surface charge [13].

Furthermore, during infection, VPg acts as a primer, analogous to the 5′ cap, being essential for both the translation and replication of viral RNA, after undergoing a process called uridylylation [14]. During uridylylation, the nuclear inclusion protein b (Nib) acts as a viral RNA polymerase and covalently links a uridine monophosphate (UMP) molecule to a specific tyrosine residue (Tyr-Y) in the VPg protein, converting it into VPg-pUpU [15,16]. These features enable the Potyvirus to infect a broad range of plant hosts, consolidating this genus as one of the most relevant for agriculture [9,17].

In addition to the responses triggered by innate immunity, also called dominant resistance, mediated by resistance (*R*) genes, plants can also exhibit recessive resistance [18]. Recessive resistance, known for the loss of susceptibility and related to susceptibility (*S*) genes, is correlated with the absence or mutations of host genes that encode proteins, essential for viral replication and life cycle [19,20].

Mutations in these genes can lead to non-synonymous substitutions in the encoded protein, thereby altering the physicochemical properties of specific amino acids [21]. Considering the perspective of their three-dimensional folding, these amino acid changes may induce local and global structural alterations in the molecule [22]. Such changes can compromise the structural stability, dynamics, and ability to interact with its natural ligands [23] and may even lead the host to manifest resistance to some pathogens. For Potyvirus, recessive resistance in several crops have been frequently associated with eIF4E factors and their isoforms [17] because the mandatory eIF4E-VPg interaction is fundamental for viral success [24].

In eukaryotes, eIF4E interacts with the 5′ cap end (m7GpppN) of mRNA. Then, it recruits the eIF4G protein and the eIF4A helicase, among other accessory subunits, ultimately forming the eIF4F complex. Once assembled, the translation process begins through ribosome recruitment [25]. Throughout plant evolution, due to duplications, mutations, and translocations, paralogs and/or isoforms of eIF4E, named eIF(iso)4E, have emerged [26,27]. These isoforms often display functional redundancy; for example, it has been demonstrated that the deletion of eIF(iso)4E in *Arabidopsis thaliana* resulted in increased expression of eIF4E, suggesting functional redundancy between these genes [28]. Furthermore, although the amino acid sequences between isoforms show moderately low identity, their three-dimensional structures remain highly conserved [29].

Even if plants possess one or more eIF4E isoforms, it has been demonstrated that Potyviruses show a preference for only one available isoform, usually the one considered primary in the translation pathway, using it as a susceptibility factor [20,30]. For example, PVY uses only one eIF4E isoform, the main one for translation, among the six available in tobacco plants [31]. In other cases, a Potyvirus may require more than one isoform as a susceptibility factor [17]. This was reported for PVY, which uses two isoforms in potato (eIF4E1 and eIF4E2) for viral mRNA translation but does not interact with eIF(iso)4E [32]. Meanwhile, *pepper veinal mottle virus* (PVMV) uses eIF4E2 in tomato species and, on the other hand, switches to eIF4E1 and eIF(iso)4E in pepper species [33,34], suggesting that the same Potyvirus may require distinct eIF4E isoforms in different crops [17].

To date, the nature of the natural recessive resistance of *V. unguiculata* to Potyvirus remains unclear. In this context, our study aimed to investigate mutations in the *eIF4E* gene in cowpea cultivars and their possible relationship with susceptibility/resistance to the Potyvirus CABMV through molecular and computational approaches. This study is essential for understanding the biology of Potyvirus infection in cowpea with impacts on the genus *Vigna*, considering both the socioeconomic importance of these legumes and the fundamental role of eIF4E/iso4E isoforms in Potyvirus infection. Moreover, the insights gained from this study provide a valuable foundation for future marker-assisted selection aiming at cowpea’s genetic improvement.

## 2. Materials and Methods

### 2.1. Analysis of the Coding Sequence of eIF4E Genes and VPg Coding Sequence

Two susceptible cowpea cultivars (cv Boca Negra and BR14 Mulato) and one resistant cultivar (cv IT85F-2687) to CABMV were evaluated for the presence of mutations in the coding sequence (CDS) of the *eIF4E* gene. Total RNA extraction was performed on leaves of 4-week-old plants (both cultivars) using the SV Total RNA Isolation System kit (Promega, Madison, WI, USA). For each of the three cultivars analyzed, three independent biological replicates were used, corresponding to samples from different plants grown under the same experimental conditions. Each RNA extraction was analyzed in technical duplicate, totaling two technical replicates per biological replicate. RNA extraction was also carried out from leaves of 4-week-old virally symptomatic plants (previously inoculated with CABMV). After DNase I treatment (Invitrogen, Carlsbad, CA, USA), RNA quality was estimated by agarose gel electrophoresis and quantified by a Qubit 2.0 Fluorometer (Invitrogen). For each sample, 1 µg of RNA was converted to cDNA using the ImProm-II Reverse Transcription Systems kit (Promega). The primer pairs 5′ATGGTTGTGGAAGATTCACAA 3′ (forward) and 5′ TCATATCACGTATTTATTTTTAGCACCC 3′ (reverse) and 5′CATATGGGGAAGAAAAGGATGATACAGAAG 3′ (forward) and 5′ CTCGAGTTCAACTCCAACATCTTCATTGGG 3′ (reverse) were used for amplification of *eIF4E* and VPg coding sequences, respectively. The total reaction volume was 20 µL, containing 1 µL of cDNA, 2 µL of 10× buffer, 0.3 µL of each primer (forward and reverse), 0.2 µL of dNTPs, 0.1 µL of Taq DNA polymerase, and 15.5 µL of H_2_O. Amplification reactions were performed using a TC-412 Thermal Cycler (Bibby Scientific, Staffordshire, UK). All samples were amplified in triplicate assays using the following conditions: 95 °C for 7 min, followed by 35 cycles at 95 °C for 1 min, 60 °C for 1 min, and 72 °C for 1 min, and a final extension at 72 °C for 10 min. The PCR band was extracted from the agarose gel, then purified with a PureLink Quick Gel Extraction Kit (Invitrogen) and cloned into the pGEM-T Easy PCR Product Cloning kit (Promega), according to the manufacturer’s instructions. Individual *E. coli* DH10B colonies (Invitrogen) were selected for plasmid preparation using the QIAprep Spin Miniprep Kit (Qiagen, Hilden, Germany) for plasmid purification. The authenticity of the CDSs was confirmed by Sanger sequencing, using the BigDye Terminator v3.1 Cycle Sequence Kit (Applied Biosystems) in a 2250XL DNA analyzer (Applied Biosystems, Foster City, CA, USA).

### 2.2. Confirmation of Mutations in the eIF4E Gene in Cowpea

The sequencing result of the *eIF4E* gene CDSs revealed the presence of mutations between the susceptible cultivars (cv Boca Negra and BR14 Mulato) and the resistant cultivar (cv IT85F-2687) to CABMV. Based on this, two pairs of primers were designed, one for each mutation type: eIF4E_Susc (F: 5′ GACCTTCTGGTTCGACAACCC 3′; R: 5′ GCAGTGAAAGTCCGCCCC 3′) and eIF4E_Res (F: 5′ GACCTTCTGGTTCGACAACCG 3′; R: 5′ GCAGTGAAAGTCCACCCG 3′). The forward sequence of the eIF4E_Susc primer recognizes position 203 with a cytosine (C203), and the reverse sequence recognizes position 325 with a guanine (G325), both positions associated with susceptibility to CABMV Potyvirus. In the eIF4E_Res primer, the forward sequence recognizes position 203 with a guanine (G203), and the reverse sequence recognizes position 325 with a cytosine (C325), potentially associated with resistance.

### 2.3. Analysis of eIF4E Gene Mutations and Assessment of Susceptibility/Resistance to CABMV in Cowpea Cultivars

The possible association of *eIF4E* gene mutation with susceptibility or resistance to CABMV was evaluated in 27 cowpea cultivars. The plants were cultivated in 4 L pots containing soil and vermiculite (1:1), under controlled greenhouse conditions at the Agronomic Institute of Pernambuco (IPA), Pernambuco, Brazil. For each cultivar, two pots were prepared, with four plants per pot. One week after sowing, leaf tissue samples were collected for DNA extraction following the method of Doyle and Doyle (1987) [35]. Plants were inoculated with CABMV as described by Oliveira and collaborators [36]. PCR reactions were performed as previously described in Section 2.1, using the primer pairs eIF4E_Res and eIF4E_Susc (annealing temperature of 55 °C and amplification product of 297 bps).

The viral inoculum was prepared from infected leaf tissues, which were macerated [0.01 M sodium phosphate buffer (pH 7.0)] at a 1:9 (*w*/*v*) ratio of tissue to buffer. For inoculation, a pestle was dipped into the viral extract and gently rubbed onto the surface of leaves (trifoliolate leaves) from cultivars. Prior to inoculation, the leaves were dusted with 600-mesh Carborundum to create micro-wounds and facilitate viral entry. After inoculation, the residual inoculum and abrasive were rinsed off with distilled water [36]. Disease symptoms were evaluated through regular inspections of the inoculated plants, by observing and recording the emergence and development of any symptom, such as vein clearing, chlorosis, or mosaic. Plants with visible symptoms were classified as susceptible and those without symptoms as resistant [37].

An additional group of plants not inoculated with CABMV was evaluated for mutation in the CDS of the *eIF4E* gene. RNA isolation, cDNA synthesis, PCR reactions, purification, and sequencing were performed as described in Section 2.1, except for the annealing temperature (57 °C), as well as the primer pair used, SeqVuELF4E–Indel (F: 5′ CTAGCAGGGTCGACAACG 3′; R: 5′ CTGAGCAGCTTCATTTGAAGC 3′), amplifying a fragment of 527 base pairs (bps). The data was processed to assess sequence quality and to exclude low-quality reads using BioEdit software (v. 7.7). Subsequently, nucleotide sequences were aligned using the ClustalW software in the MEGA package (v. 11) and visualized with Jalview (v. 2.11) [38]. These sequences were analyzed alongside those from the three cultivars initially examined in the study—Boca Negra, BR14 Mulato, and IT85F-2687, which served as reference standards for comparative analysis.

### 2.4. Primary Sequences, Alignments, and Conserved Domain of eIF4E Coding Region

To investigate the possible impact of specific mutations in the *eIF4E* gene on its interaction with VPg (CABMV)—a factor potentially associated with susceptibility or resistance to the virus—five cowpea cultivars (Bajão, Boca Negra, BRS Cauamé, BRS Xiquexique, and IT85F-2687) were selected for in silico analyses. This selection was based on mutations in the *eIF4E* coding sequence (CDS) and bioassay results. Boca Negra and BRS Xiquexique were both susceptible to CABMV, although BRS Xiquexique carries a thymine at position 325. BRS Cauamé and IT85F-2687 share mutations at positions 203 and 325, but only IT85F-2687 showed a resistant phenotype. Bajão exhibited unique changes, including a six-base-pair insertion between positions 223 and 230. The nucleotide sequences of these cultivars were evaluated via BLAST (v.2.16.0) https://blast.ncbi.nlm.nih.gov/Blast.cgi (accessed on 10 July 2024 to confirm the conservation of the *eIF4E* gene. Subsequently, the primary sequences of the eIF4E proteins (from the five cultivars, along with the VPg protein) were translated using the ORFfinder tool https://www.ncbi.nlm.nih.gov/orffinder/ (accessed on 10 July 2024. The search for functional domains of the proteins was performed via CD Search https://www.ncbi.nlm.nih.gov/Structure/cdd/wrpsb.cgi (accessed on 11 July 2024). In parallel, a search for VPg protein sequences from different *Potyviruses* was conducted on NCBI https://www.ncbi.nlm.nih.gov/ (accessed on 14 July 2024) to verify conserved domains (Figure S1). All sequence alignments were performed using the ClustalW method in the MEGA package (v. 11) and subsequently visualized in Jalview (v. 2.11).

### 2.5. Molecular Modeling, Model Validation, and Molecular Dynamics Simulations

The predicted theoretical models were selected based on two confidence metrics, the Predicted Local Distance Difference Test (pLDDT) and Predicted Aligned Error (PAE), both available in the AlphaFold 3 algorithm [39]. The models were validated for folding quality and thermodynamics using ProSA, PROCHECK, and QMEANDisCo. Furthermore, the theoretical models were aligned with an experimental model from *Arabidopsis thaliana* (PDB ID: 5BXV). Molecular dynamics (MD) simulations for the isolated eIF4E, the VPg proteins, and the eIF4E-VPg complexes (obtained by molecular docking with HADDOCK) were analyzed and executed in the GROMACS package [40]. Physiological conditions were simulated at 0.15 M, with water (SPC type) and NaCl ions, for 100 ns, using the GROMOS 53A6 force field [40,41]. Periodic boundary conditions were applied in the x, y, and z directions, centering the models in a cubic box of 10 nm × 10 nm × 10 nm, followed by energy minimization of the systems [40,42]. In the NVT step, the temperature was set to 300 K, employing solute atom restraints at the initial positions [40]. The LINCS method was used to apply constraints to the covalent bonds of the systems, including those involving hydrogen atoms [43]. Integration was performed by the leapfrog algorithm, using an integration time step of 2 fs [44]. Using the steepest descent algorithm, the energy of the systems was optimized using 50,000 steps. MD simulations were performed without restraints, with constant temperature and pressure (300 K and 1 atm, respectively) for 100 ns. The MD simulation trajectories were also analyzed with GROMACS, considering the following parameters: Root Mean Square Deviation (RMSD), Root Mean Square Fluctuation (RMSF), radius of gyration (RG), and hydrogen bonds (HBs) [40]. Finally, Electrostatic Surface Potential (ESP) analyses were performed using the APBS server [45].

### 2.6. Molecular Docking and Binding Energy Between eIF4E-VPg Complexes

Molecular docking was performed with representative models from each trajectory of the isolated systems simulated by MD. The molecular docking interaction between the proteins of the selected cultivars, eIF4E and VPg, was submitted to HADDOCK (v. 2.4), following the software’s default settings. Interaction residues were defined according to the literature [13]. For VPg, the residues determined as active were F107, I108, E112, S115, Q116, and V118. For the eIF4E proteins of Boca Negra, IT85F-2687, BRS Cauamé, and BRS Xiquexique, they were W22, W68, R118, R120, and K123. In contrast, for the eIF4E protein of the Bajão cultivar, the residues were W24, W70, R120, R122, and K125. The pairs generated by molecular docking were selected based on the most favorable HADDOCK score values, considering the combination of cluster positions and physicochemical characteristics of the interactions. The results were submitted to the Rosetta package (v. 4.0) to calculate the Gibbs free energy between eIF4E and VPg. Additionally, the theorical binding free energy for each complex was estimated using the MM/GBSA method. For this purpose, all complex structures were submitted to the HawkDock server, defining eIF4E (chain A) as the receptor and VPg (chain B) as the ligand [46].

The complexes generated by HADDOCK were subjected to MD (parameters from Section 2.5) to assess possible alterations in eIF4E after interaction with VPg. Furthermore, the positions of the mutations identified in the five cowpea cultivars were analyzed to determine whether they are located in or close to the VPg-binding pocket.

## 3. Results

### 3.1. Mutations in the eIF4E Gene of Vigna Unguiculata

Analysis of the *eIF4E* gene revealed five mutations (at positions 203, 224, 325, 329, and 520) between the two susceptible cultivars (Boca Negra and BR14 Mulato) and the resistant cultivar (IT85F-2687) (Table 1). Of these, three mutations (at positions 203, 325, and 329) differed between the two susceptible cultivars and the resistant one (Table 1). The substitutions included the following: C203G, leading to a change from proline to arginine (P68R); G325C, with a change from glycine to arginine (Gly109Arg); and C329T, where an alanine was replaced by a valine (Ala110Val) (Table 1). At positions 224 and 520, the susceptible cultivars exhibited different types of substitutions compared to the resistant one: C224A in the Boca Negra cultivar and T520A in the BR14 Mulato cultivar.

### 3.2. Susceptibility/Resistance to CABMV Based on Mutations in the eIF4E Gene

Based on the mutations at positions 203 and 325 (observed in the *eIF4E* gene in the cultivars Boca Negra, BR14 Mulato, and IT85F-2687), two primers pairs (eIF4E_Susc and eIF4E_Res) were designed and employed in PCR assays across 27 cowpea cultivars. The eIF4E_Susc primer recognizes the C203/G325 mutations associated with susceptibility, while the eIF4E_Res primer recognizes the G203/C325 mutations associated with resistance. Of the 27 cultivars evaluated, 25 (92.52%) confirmed the amplification of the expected 297 bp fragment (Figure S2). Of these, 17 cultivars (62.96%) amplified with the primer pair for susceptibility (eIF4E_Susc: C203/G325), and eight cultivars amplified with the primer pair for resistance (eIF4E_Res: G203/C325). Notably, two cultivars (TVU-966 and Bajão) failed to amplify with either primer set.

### 3.3. Bioassay of Cowpea Cultivars Inoculated with Potyvirus CABMV

**Figure 1:**
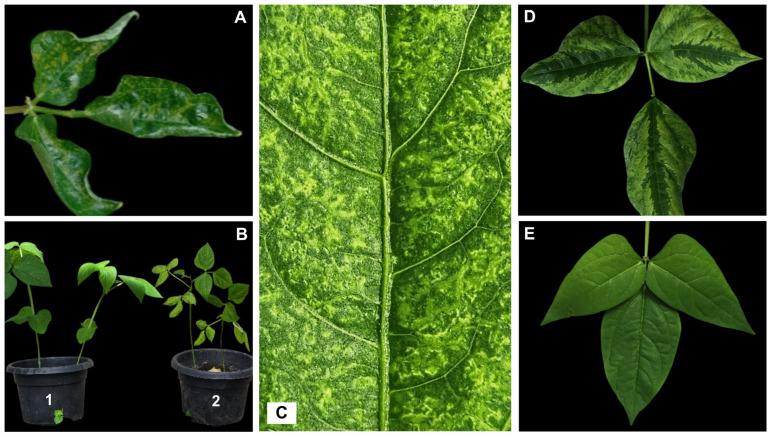
Representative symptoms exhibited by the cowpea cultivars inoculated with *cowpea aphid-borne mosaic virus* (CABMV): (**A**) Leaf distortions and mosaic symptoms. (**B**) Comparison of cowpea cultivars: (**B1**) asymptomatic plant and (**B2**) infected plant showing mosaic symptoms and reduced leaf size. (**C**) Severe mosaic. (**D**) Mosaic and green stripes along the veins. (**E**) Healthy leaf from a resistant cultivar (IT85F-2687).

To confirm the association of mutations with susceptibility/resistance to CABMV, the 27 cowpea cultivars previously analyzed by PCR were monitored for symptom development following CABMV inoculation. Of the 17 cultivars that tested positive (via PCR) for the mutation associated with susceptibility, 15 (88.24%) exhibited symptoms 10 days after inoculation (dais) (Figure 1; Table 2). Among the eight cultivars that were used for the PCR search of mutations associated with resistance, five (62.5%) remained asymptomatic, while three (37.5%) developed symptoms (Table 2). Although the cultivars TVU-966 and Bajão did not amplify with the tested primers, they present no symptoms in the bioassay, confirming their resistance to CABMV (Table 2). Figure 1 shows the main symptoms observed in the cowpea cultivars after viral inoculation, including reduced plant size and leaf distortion (Figure 1A,B), severe mosaic (Figure 1C), and mosaic and green stripes along the veins (Figure 1D).

### 3.4. Genetic Variation in the eIF4E CDS Among 27 Cowpea Cultivars

In addition to PCR amplification and symptom evaluation, plants of the 27 cultivars (not inoculated with CABMV) were also analyzed for mutations in the coding sequence of the eIF4E gene. For this purpose, the 527 bp fragments amplified from the cDNA of all 27 cultivars were sequenced. Sequence analysis revealed a high level of conservation (>98%; Figure S3), although some mutations were detected (Figure S4).

Among the 17 cultivars whose loci were amplified with the eIF4E_Susc primer pair (indicating possible susceptibility), 16 (94%) exhibited a cytosine at position 203 (C203), consistent with the expectation for susceptible phenotypes. Only one cultivar (Sempre Verde Salgueiro) displayed guanine at this position (G203). Regarding the nucleotide at position 325 (identified as G325 in susceptible cultivars), only two cultivars (BRS Xiquexique and BRS Paraguaçu, both susceptible) exhibited a thymine at this position (T325). Among the eight PCR-positive cultivars for the eIF4E_Res primer (suggesting possible resistance), all exhibited a guanine at position 203 (G203) and a cytosine at position 325 (C325). Only two cultivars failed to amplify with both primer pairs, TVu-966 and Bajão (both resistant). They exhibited nucleotide substitutions preventing the annealing of primers. These substitutions included C328/329 in the TVu-966 cultivar. Notably, the mutation at position 328 is also found in the Inhuma and Sempre Verde Salgueiro cultivars (Figure S4). Interestingly, the Bajão cultivar displayed a greater number of differences in the *eIF4E* gene compared to the other cultivars. This cultivar had the G203 mutation, also observed in other cultivars possibly resistant to CABMV. Another difference was a 6 bp insertion detected between positions 223 and 230. Furthermore, at position 331, this cultivar showed an adenine (A331), distinguishing it from the other possibly resistant cultivars (Figure S4). The mentioned mutations, classified as non-synonymous, alter the amino acid sequences of the eIF4E protein. The corresponding alterations in the protein sequence are shown in Figure S5.

### 3.5. Alignment and Conserved Domain of eIF4E and VPg Proteins

**Figure 2:**
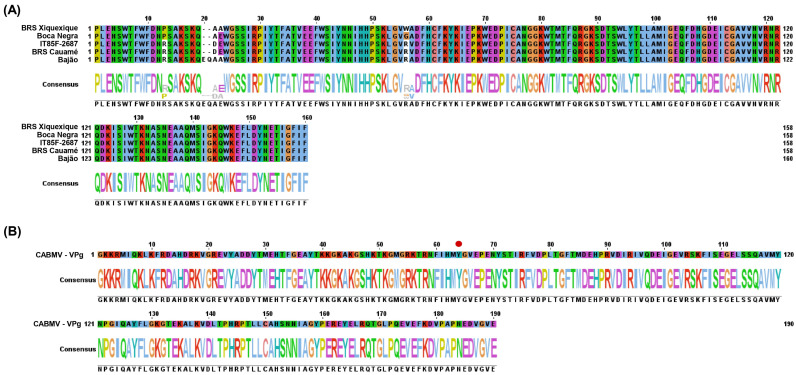
Conserved domain analysis. (**A**) Conserved domain of the eIF4E protein from five cowpea cultivars (Bajão, Boca Negra, BRS Cauamé, BRS Xiquexique, and IT85F-2687). (**B**) Conserved domain of the VPg protein from CABMV (*cowpea aphid-borne mosaic virus*). The red circle (position 64) in the VPg sequence highlights the tyrosine (Tyr) residue essential for the VPg uridylylation process.

In silico analyses were performed on five selected cowpea cultivars (Bajão, Boca Negra, BRS Cauamé, BRS Xiquexique, and IT85F-2687), based on the identified amino acid substitutions and the performance observed in the bioassay following CABMV inoculation (Table 2 and Section 3.3). These substitutions are present in the conserved domain of the cowpea eIF4E protein, as shown in Figure 2A. Figure 2B shows the conserved domain of the CABMV VPg protein.

### 3.6. Molecular Modeling and Structural Validation of eIF4E and VPg Models

The theoretical models of the eIF4E proteins from cowpea cultivars (Boca Negra, BRS Xiquexique, BRS Cauamé, IT85F-2687, and Bajão) as well as the VPg protein from CABMV, all obtained through modeling using AlphaFold 3, exhibited validation metrics within quality criteria [51,52]. Analyses performed in ProSA showed Z-Score values ranging from −6.14 to −6.63 for the three-dimensional models of eIF4E and of −4.75 for the VPg model. Furthermore, values obtained in PROCHECK revealed values above 92% for residues that were located in regions with thermodynamically favorable torsion angles. The highest scores (>95%) were observed for the VPg and the eIF4E models of Boca Negra and IT85F-2687. The models for BRS Cauamé and BRS Xiquexique exhibited scores above 93%, while the model for the Bajão cultivar had a score of 92.5%. QMEANDisCo analysis further supported the quality of the models, with all structures presenting values ranging from 0.85 to 0.87 for eIF4E and of 0.56 for VPg, highlighting the quality of the models [53].

**Figure 3:**
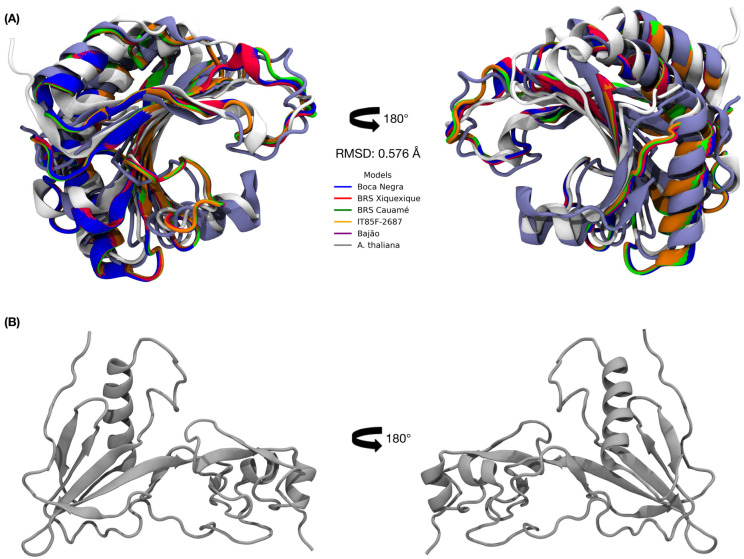
Alignment between the theoretical three-dimensional models of eIF4E proteins. (**A**) Alignment of the three-dimensional structures of cowpea eIF4E proteins with *A. thaliana* eIF4E, RMSD of 0.576 Å (PYMOL v.3.1). (**B**) Three-dimensional structure of the VPg protein from the CABMV *Potyvirus*.

The alignment of the three-dimensional models of the cowpea eIF4E proteins revealed an RMSD of 0.071 Å (Figure S6). In turn, the alignment between the cowpea eIF4E models and the *A. thaliana* eIF4E models exhibited an RMSD of 0.576 Å (Figure 3A).

**Figure 4:**
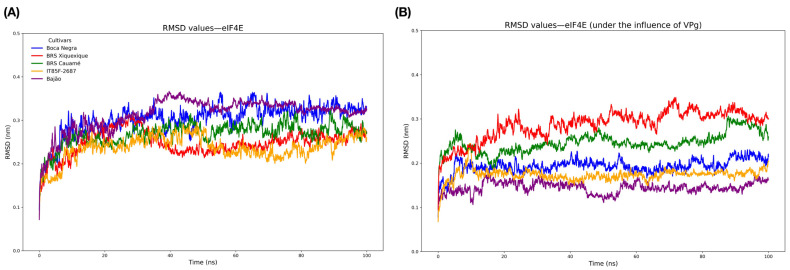
Evaluation of the structural convergence of *V. unguiculata* eIF4E proteins. (**A**) RMSD of isolated eIF4E proteins; (**B**) RMSD of eIF4E proteins after docking with VPg.

Analysis of the molecular dynamics (MD) trajectories of the five theoretical three-dimensional models of eIF4E revealed RMSD values converging in all conditions around 15 ns (Figure 4A). The BRS Xiquexique and IT85F-2687 models showed the lowest RMSD values, with structural convergence in the trajectory at 25 ns and 50 ns, respectively. In contrast, the highest RMSD values, and therefore the largest fluctuations, were observed in the Bajão and Boca Negra models, followed by the BRS Cauamé model. Additional MD results for the VPg protein are presented in Figure S7.

Data extracted from the MD trajectories indicated that the interaction of eIF4E proteins with VPg (eIF4E-VPg) caused local and global alterations in the structural dynamics of eIF4Es (Figure 4B). The eIF4E proteins from Bajão and BRS Xiquexique exhibited the largest fluctuations, as indicated by RMSD values (Figure 4B). The eIF4E protein from the Bajão cultivar, followed by that of IT85F-2687, showed less perturbation at the 5’cap-binding site in response to VPg binding. In contrast, eIF4E from BRS Xiquexique displayed global perturbations upon interaction with VPg (Figure 4B).

**Figure 5:**
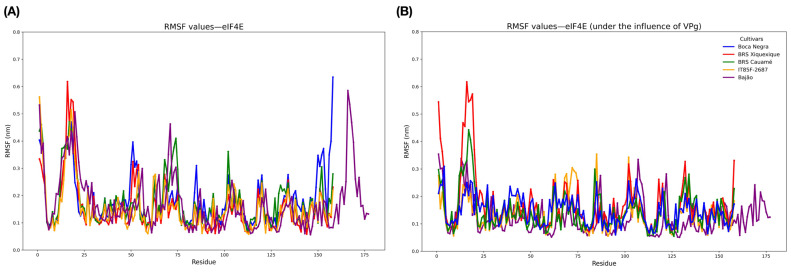
Evaluation of the flexibilities of the three-dimensional structures of *V. unguiculata* eIF4E. (**A**) RMSF of isolated eIF4E proteins. (**B**) RMSF of eIF4E proteins after docking with VPg.

Additionally, RMSF analysis revealed similar flexibility profiles among the systems before binding with VPg (Figure 5A), with emphasis on BRS Cauamé and Bajão, which displayed an exclusive flexibility peak between residues 65 and 78. Boca Negra exhibited a fluctuation peak at residue 150, while Bajão showed a characteristic peak between residues 150 and 175, differing from the other evaluated systems. The degree of flexibility of each region in the protein structures is visualized in Figures S8 and S9. Notably, the 160–175 region of eIF4E showed a marked decrease in flexibility upon VPg binding (Figure S8). This stabilization suggests the direct involvement of this segment in the binding interface. This result aligns with previous reports that map the interaction site to the C-terminal domain of eIF4E [54,55]. Following VPg interaction (Figure 5B), the RMSF profiles of the eIF4E proteins showed a global reduction in flexibility across all cultivars. However, the BRS Cauamé and BRS Xiquexique models exhibited an exclusive gain in flexibility between residues 8 and 16 (Figure 5B).

**Figure 6:**
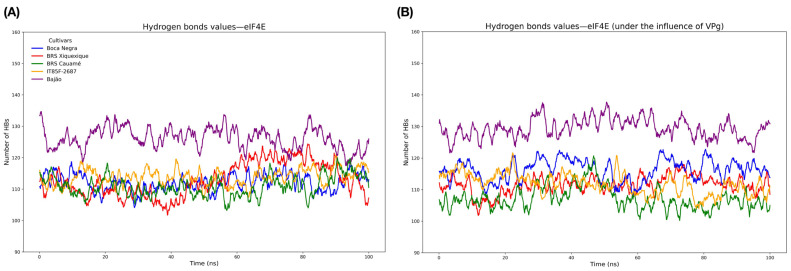
Evaluation of the intramolecular hydrogen bonds (HBs). (**A**) Number of intramolecular HBs in isolated eIF4E proteins; (**B**) number of intramolecular HBs after interaction with the VPg protein (eIF4E-VPg).

In addition to structural convergence and flexibility analyses, we also investigated properties related to protein compaction and secondary structure maintenance, using the number of intramolecular hydrogen bonds (HBs) and radius of gyration (RG) as parameters. HB analysis revealed that the eIF4E protein of the Bajão cultivar exhibited the highest number of HBs, whereas the other cultivars showed similar values (Figure 6A). Furthermore, the number of HBs remained unchanged following the eIF4E-VPg interaction (Figure 6B).

**Figure 7:**
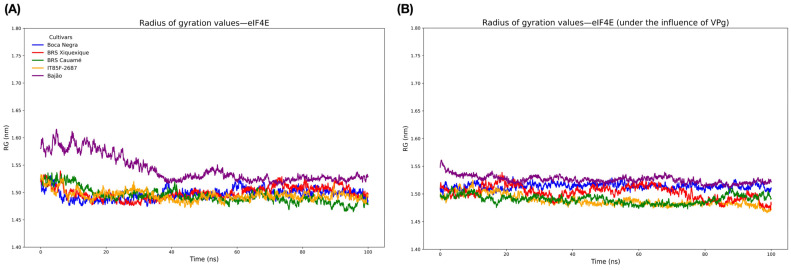
Evaluation of the radius of gyration (RG) of eIF4E proteins. (**A**) RG of isolated eIF4E proteins; (**B**) RG after docking with VPg (eIF4E-VPg).

Regarding the degree of compaction, as assessed by the radius of gyration (RG) parameter of the proteins, the eIF4E protein isolated from the Bajão cultivar showed lower compaction during the first 40 ns but then behaved similarly to the other cultivars throughout the trajectory. For the eIF4E-VPg complex, all cultivars displayed a numerically similar degree of compaction among themselves (as also observed when analyzed in isolation) (Figure 7).

**Figure 8:**
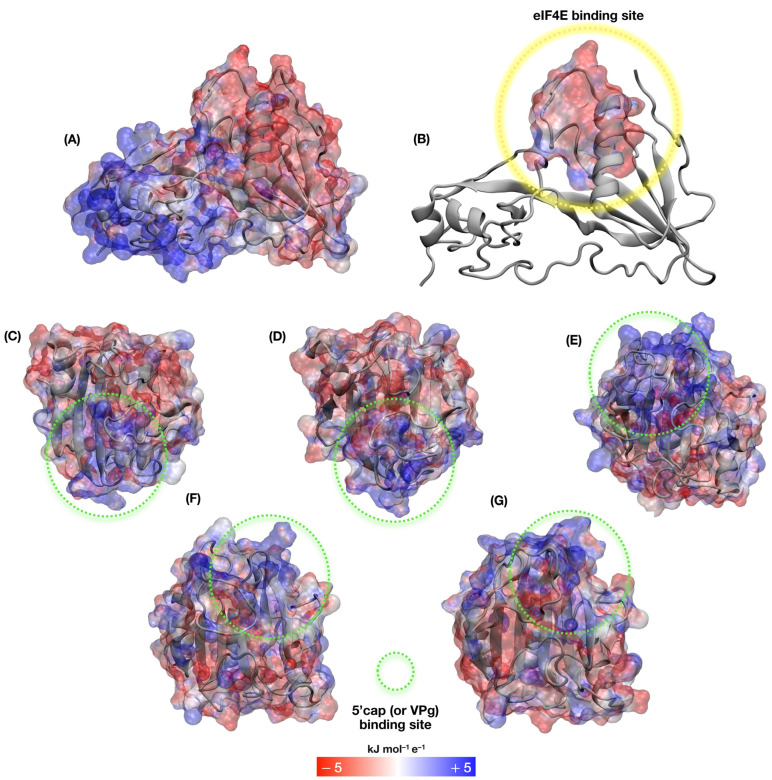
Evaluation of the electrostatic surface profiles of eIF4E structures from cowpea cultivars and the VPg protein from CABMV Potyvirus. Dots highlight the eIF4E-VPg interaction regions with anionic (green circle) and cationic charges (yellow circle), respectively. (**A**) VPg; (**B**) VPg’s eIF4E binding site; (**C**) Boca Negra; (**D**) IT85F-2687; (**E**) Bajão; (**F**) BRS Xiquexique; (**G**) BRS Cauamé.

To understand the electrostatic contribution of the eIF4E-VPg interaction, we evaluated the surface charge distribution of eIF4E proteins. Possible alterations in protein charges, due to the observed mutations in the eIF4E of cowpea cultivars, were also assessed. This analysis revealed opposite charge profiles localized in different VPg regions. The region directly interacting with eIF4E exhibited a predominantly anionic electrostatic profile (Figure 8A,B), whereas another site displayed strongly cationic characteristics (Figure 8). The VPg interaction site (dedicated to mimicking the 5′ cap) showed predominantly negative charges (Figure 8A,B). The data also revealed that the region responsible for recognizing and binding to the 5’cap of mRNA in eIF4E is cationic in nature.

### 3.7. Molecular Docking, Interface Analysis, and Binding Energy

The docking scores for the eIF4E-VPg complexes, which were generated using the previously described AlphaFold 3 theoretical models and docked with HADDOCK, ranged from −72.3 to −115.5 (Table 3). The most favorable interactions (indicated by more negative scores) were observed between VPg and eIF4E from the BRS Xiquexique (−102.9) and IT85F-2687 (−115.5) cultivars. In contrast, dockings with eIF4E from the BRS Cauamé and Bajão cultivars obtained the highest scores (−72.3 and −75.9, respectively) and were, therefore, less favorable for interaction with CABMV VPg.

**Figure 9:**
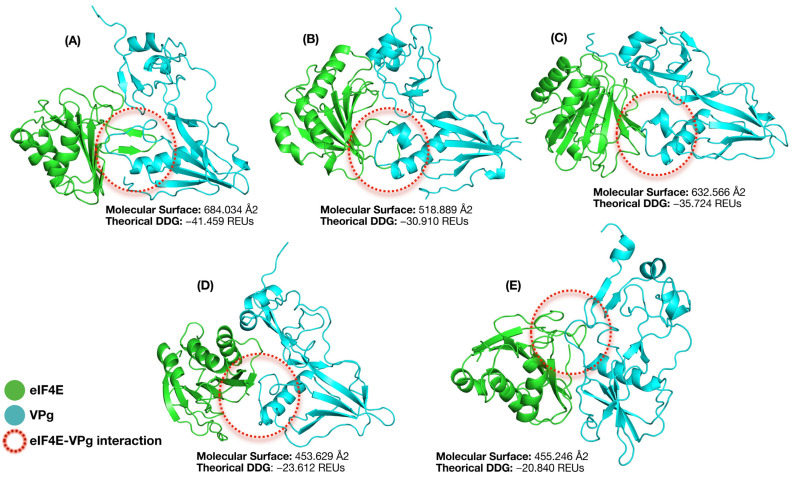
Schematic representation of the interaction areas in Å2 of the VPg-eIF4E complex and the corresponding Gibbs free energy difference (Theorical DDG), expressed in Rosetta Energy Units (REUs). (**A**) Boca Negra; (**B**) IT85F-2687; (**C**) Bajão; (**D**) BRS Xiquexique; (**E**) BRS Cauamé.

Additionally, the free binding energy and interaction area between the proteins were calculated with the Rosetta package, aiming to understand the eIF4E-VPg fit (Table 3). It should be noted that the absolute values of these computationally derived energies carry inherent uncertainty. Therefore, it is more robust to use them to establish relative energetic trends, for which the susceptible Boca Negra complex was used as the baseline to compare the other cultivars. The Boca Negra cultivar showed the largest interaction area, as well as the most favorable binding energy among all complexes tested. The BRS Cauamé and BRS Xiquexique cultivars showed the smallest interaction areas between the eIF4E-VPg complex, as well as the lowest binding energies (Figure 9).

Furthermore, the binding free energies calculated using the MM-GBSA method through HawkDock confirmed the energetic trends predicted by the other two previously presented tools (Table 3). Notably, in addition to the total binding free energy, this analysis also provided per-residue energy decomposition, highlighting the key residues that contribute to the stability of the eIF4E-VPg complex. This decomposition detailed the specific contributions from van der Waals (VDW), electrostatic (ELE), polar solvation (GB), and nonpolar solvation (SA) interaction components. The per-residue energy decomposition (Supplementary Table S1) revealed a conserved interaction “*hotspot*” in the 118–121 region of eIF4E, where residues such as Arg-118 and Arg-120 act as fundamental binding anchors in most variants, underscoring the importance of this region for the formation of the eIF4E-VPg complex. A clear pattern emerged from this hotspot: it is dominated by a strong positive charge, creating a key electrostatic surface. The analysis also highlighted the diversity in binding strategies: the BRS Xiquexique cultivar presented a high interaction dominated by Arg-120 (exhibiting a predicted theoretical binding free energy of the complex = −14.89 kcal/mol), as exhibited in Table S1, while the Bajão cultivar demonstrated a divergent recognition pattern, utilizing a distinct set of residues at the interface (e.g., Pro-73, Ile-74).

Notably, the Pro68Arg substitution stood out as the only one among the central mutations of this study to directly and significantly impact the binding energy at the interface, as evidenced by its high contribution in the Boca Negra cultivar. This finding, along with the relevance of the Trp-68 residue in the same system, demonstrates the importance of electrostatic and aromatic interactions at the main eIF4E interface (cap and VPg binding). In contrast, the other analyzed mutations are not among the main energetic contributors, suggesting their role in resistance is likely indirect, acting through the modulation of the protein’s global conformation and dynamics, a hypothesis supported by our MD analyses. The extended per-residue decomposition results for all systems are available in Supplementary Table S1.

These MM-GBSA-based findings help clarify the molecular recognition process between the systems, offering important clues as to the mechanism of action for the investigated resistance mutations. However, it is important to note that the absolute binding energy values derived from computational methods carry significant uncertainty, largely due to the challenge of accurately capturing the entropic component of binding. Therefore, these values are best interpreted not for their absolute magnitude but as semi-quantitative estimates that reliably indicate the energetic trends and relative differences between the systems.

**Figure 10:**
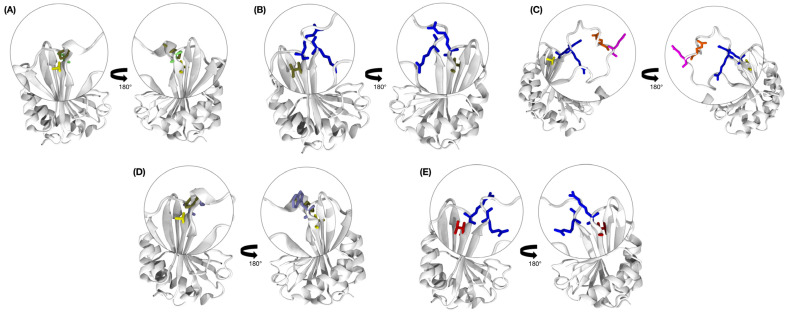
Highlighted representation of arginine (blue), glutamate (orange), glutamine (magenta), alanine (yellow), proline (tan), glycine (lime), valine (red), and tryptophan (purple) residues in *V. unguiculata* in the VPg-binding pocket of eIF4E proteins. (**A**) Boca Negra; (**B**) IT85F-2687; (**C**) Bajão; (**D**) BRS Xiquexique; (**E**) BRS Cauamé.

The Boca Negra cultivar exhibited the largest interaction area, as well as the most favorable binding energy, among all complexes analyzed. Interestingly, the resistant cultivars IT85F-2687 and Bajão displayed intermediate interaction surface areas and free binding energies when compared to the other cultivars. Three-dimensional models of eIF4E revealed that the mutations identified in this study are located within the VPg-binding pocket (Figure 10).

Complementarily, other strategies for identification of key amino acid residues driving the eIF4E-VPg interaction were accomplished through two distinct analyses of the docking-derived MD trajectories. Initially, the most representative structure was selected from each of the five eIF4E-VPg systems, one for each cultivar. In this analysis, these structures were used to map intermolecular contacts by categorizing all eIF4E residues located within a 5 Å radius of VPg (Supplementary Tables S2–S7). Concurrently, hydrogen bond occupancy was quantified across these MD trajectories, applying strict geometric criteria (donor–acceptor distance ≤ 3.5 Å and angle ≥ 120°) to identify the most persistent interactions stabilizing the eIF4E-VPg interface (Supplementary Tables S8–S12).

A combined analysis of the top favorable energetic residue contributions from MM-GBSA (Supplementary Table S1), the frequency of all interaction types (Supplementary Tables S2–S7), and hydrogen bond occupancy (Supplementary Tables S8–S12) revealed a set of key residues at the eIF4E-VPg-binding interface. Among the aromatic residues, Trp-68 was a standout (Supplementary Tables S1 and S7), not only ranking as the top energetic contributor for the Boca Negra cultivar but also being one of its most frequent interactors. This trend was also observed for other hydrophobic residues, such as Ile-74 and Pro-73 in the Bajão cultivar, as shown in Supplementary Tables S1 and S7. The analysis underscored the role of positively charged residues, particularly Arg-118, which was identified as a convergent “*hotspot*” in four (Boca Negra, BRS Cauamé, BRS Xiquexique, and IT85F-2687) of the five cultivars studied. Other charged residues, such as arginine and lysine, also showed a correlation between high energetic contribution and frequent contact (Supplementary Tables S1 and S7). Furthermore, polar uncharged residues were found to be essential, exemplified by Gln-121, which was the most significant energetic contributor for BRS Cauamé while also being a frequent binding interactor.

In addition, the hydrogen bond occupancy analysis (Supplementary Tables S8–S12) highlighted the key interaction anchors by quantifying the stability of the most persistent bonds throughout the MD trajectories. For the BRS Cauamé cultivar, a persistent stable hydrogen bond (97.36% occupancy) was observed between eIF4E’s backbone carbonyl oxygen of Trp-68 and VPg’s amide nitrogen of Gly-111, suggesting this interaction serves as a fundamental structural lock. In Boca Negra and IT85F-2687, the charged residue Arg-120 was central, forming persistent hydrogen bonds (51.55% and 46.58%, respectively), highlighting the role of electrostatic forces in complex stabilization. In the Bajão cultivar, the most durable interaction (62.34%) occurred between the backbone (Gly-25) and the polar side chain of Ser-115 (VPg), indicating a combination of specificity and structural stability. In contrast, the BRS Xiquexique cultivar displayed more transient hydrogen interactions, with the highest occupancy (Lys-18 and Gly-111) reaching only 4.60%, which may correlate with a different binding affinity profile.

Taken together, these results of correlation between the binding scores, per-residue energetic contribution, and contact frequency, enriched by hydrogen bond occupancy data, provide robust evidence for identifying the most functionally relevant residues at the eIF4E-VPg interface. The consistent identification of residues like Arg-118, Trp-68, and Gln-121 and the importance of H-bond anchors involving Arg-120 and backbones suggest that the interface’s stability relies on a cooperative network of hydrophobic, electrostatic, and hydrogen-bonding interactions, the specific balance of which dictates the affinity in each cowpea cultivar.

## 4. Discussion

Recessive resistance of plants to Potyvirus infection can be acquired through natural (or induced) mutations in the eIF4E protein and its isoforms (eIF (iso) 4E). This type of resistance typically results from single-nucleotide polymorphisms (SNPs) [56], involving non-synonymous amino acid substitutions, which can hinder viral infection, reducing symptoms in the host plant [57]. Based on this mechanism, the present study aimed to investigate the effect of mutations in the coding region of the *eIF4E* gene and their potential role in determining susceptibility or resistance of selected cowpea cultivars to *cowpea aphid-borne mosaic virus* (CABMV).

Five non-synonymous mutations in the *eIF4E* gene were identified among the three cowpea cultivars analyzed in this study. Of these, three mutations (at positions 203, 325, and 329) distinguish the two susceptible cultivars (Boca Negra and BR14 Mulato) from the resistant cultivar (IT85F-2687). These mutations corresponded to the nucleotide substitutions C203G, G325C, and C329T, resulting in amino acid changes Pro68Arg, Gly109Arg, and Ala110Val, respectively. The first two mutations involve the replacement of apolar hydrophobic amino acids (proline and glycine) with arginine, a basic amino acid with a positive charge, resulting in important structural and functional changes in the eIF4E protein. On the other hand, the third mutation (Ala110Val), although also non-synonymous, involves a substitution between two apolar amino acids, (hydrophobic), not altering the charge or polarity of the protein [58]. It is worth noting that point mutations can lead to local and global alterations in the protein and, consequently, affect its interaction with other proteins [59], such as VPg. These alterations may contribute to resistance against Potyvirus infection. Similar substitutions, at the same or nearby positions in eIF4E, have previously been associated with recessive resistance to Potyviruses [56]. Amino acid substitutions such as Val67Glu, Ala68Glu, and Gly107Arg have been identified in pepper resistant to *potato virus Y* (PVY) [34,60]. Similarly, in lettuce and watermelon, resistance to Potyvirus has been associated with the Arg107Gly and Asp71Gly substitutions, respectively [61,62]. These mutations reflect a pattern in which basic or acidic amino acids (Arg, Glu, or Asp) found in resistant cultivars were replaced by apolar amino acids (Gly, Ala, or valine) in susceptible phenotypes, consistent with the pattern observed in cowpea in the present study.

Molecular approaches, including PCR amplifications using specific primers for each mutation (resistant/susceptible), largely supported the results from the viral infection assay across the 27 cowpea cultivars. Similarly, sequencing and alignment results confirmed the presence of mutations at positions 203 and 325 in most of the cultivars tested.

While BRS Tumucumaque, BRS Cauamé, and BRS Guariba have been described as susceptible [48,49], other studies have reported BRS Cauamé and BRS Guariba as resistant to CABMV [47]. The discrepancy observed (where some cultivars amplified with the primer associated with resistance but showed symptoms of CABMV) may be explained by resistance breakdown. Such breakdowns are often driven by viral mutations, as observed in the coat protein (CP) of *Pepino mosaic virus* (PepMV) [63]. Resistance breakdown mediated by eIF4E occurs mainly through single or multiple mutations in the VPg protein, allowing the virus to reuse eIF4E or another isoform as a susceptibility factor [64], as reported in pepper, tomato, pea, and barley [64,65]. These alterations represent one of the main factors responsible for the decrease in the durability of recessive resistance of plants to phytopathogens [20], such as Potyvirus. Furthermore, some plants, when possessing resistant *eIF4E* alleles in homozygosity, may occasionally exhibit symptoms, albeit late [66,67].

In the case of the cultivars Miranda IPA 207 and Manteiguinha Santarém, although both tested PCR-positive for susceptibility, they did not show symptoms after viral infection. Notably, the Miranda IPA 207 cultivar has previously been described as susceptible, whereas Manteiguinha Santarém has been reported as resistant to CABMV [48]. The latter, Manteiguinha Santarém, could be a target for future studies aimed at prospecting genes related to cowpea resistance to CABMV. Moreover, understanding the involvement of susceptibility genes in the plant–virus pathosystem is fundamental for advancing genetic improvement programs focused on developing resistant cultivars.

The alignments of the three-dimensional structures revealed a high conservation of the three-dimensional folding of eIF4E among the five cowpea cultivars, corroborating the data described experimentally in several studies [13]. Despite this, some point mutations were observed, which can cause local and global alterations in the protein [59,68,69]. These mutations are close to the eIF4E binding site with VPg (Figure 10), suggesting they could potentially affect their interaction.

Among the five proteins analyzed in silico, BRS Xiquexique and Bajão showed the most pronounced discrepancies in RMSD values. The Trp109Gly substitution in BRS Xiquexique, which distinguishes it from the other susceptible cultivars, and the addition of two amino acid residues (Glu and Gln) at positions 76 and 77, respectively, in Bajão, may account for the observed RMSD deviation. These findings suggest that BRS Xiquexique and Bajão presented the greatest alterations throughout the MD. When interacting with VPg, the eIF4E of Bajão was less perturbed, unlike BRS Xiquexique, which exhibited global perturbations after the interaction. These observations may be associated with viral resistance mechanisms, as CABMV relies critically on hijacking eIF4E, a key component of the translational complex to complete its viral replication process [17,20,67]. Moury et al. [33] demonstrated that knocking out the *eIF4E* gene in tomato conferred resistance to *pepper veinal mottle virus* (PVMV), proving the importance of *eIF4E* genes in Potyvirus infection.

The interaction with VPg markedly altered the flexibility profile of the eIF4E proteins in the analyzed cultivars, suggesting that the formation of the eIF4E-VPg complex generates global conformational charges. These alterations may result from steric hindrances due to proximity to VPg or from conformational adjustments directly caused by the interaction. The fluctuation peak observed between residues 8 and 16 (characteristic of the BRS Xiquexique and BRS Cauamé cultivars) coincided with the site of the Pro68Arg mutation, differentiating susceptible cultivars from resistant ones. Nearby residues may have led to increased flexibility, since the properties of amino acids have the potential to influence protein flexibility significantly [70,71]. A remarkable contribution to the entropy of a system consists of its greater flexibility which, in turn, interferes with interactions with other systems [72]. This observation supports the experimental data, as BRS Cauamé, despite presenting mutations similar to those found in resistant cultivars, exhibited a flexibility peak comparable to that of the susceptible BRS Xiquexique cultivar and behaved as susceptible when inoculated with CABMV.

Moreover, our findings shed light on the dynamics of the N-terminal region of eIF4E. In agreement with the previous literature [73,74], our MD simulations suggest that this domain (residues 1–25) is highly flexible, as indicated by its high RMSF values (Figure 5), and that its flexibility is not completely reduced upon VPg binding (Figure 5 and Figure S8). This is consistent with the established role of the N-terminal domain of eIF4E as the primary binding site for eIF4G, a crucial interaction for the initiation of translation hijacked by Potyviruses [74,75]. This raises an intriguing possibility for the mechanism of the Pro68Arg resistance mutation. Rather than directly impeding the VPg interaction, the Pro68Arg substitution could remotely modulate the dynamics of the N-terminal domain. Such a long-range effect could consequently impair the recruitment of eIF4G, disrupting viral polyprotein synthesis and ultimately leading to the resistant phenotype. This proposed structural pattern mechanism provides an interesting hypothesis for future experimental validation.

Although eIF4E factors play a critical role in viral replication and contribute to recessive resistance in plants, other viral proteins may also act as secondary factors influencing susceptibility or resistance. For example, the HC-Pro protein has been shown to interact with eIF4E isoforms in peanut [76], in addition to VPg. The interaction of HC-Pro, VPg, and eIF4E is essential not only for the efficient translation of viral RNA [76] but also for the suppression of the host plant’s defense responses [77].

Intramolecular HBs play a fundamental role in various biological processes, including the stabilization of three-dimensional protein structures [78]. In addition to HBs, the conformation and compaction of protein structures analyzed through RG [79] is an important parameter for MD studies. The results obtained from HB and RG analysis indicate that the eIF4E-VPg interaction does not affect the degree of compaction, which was numerically similar among them and also like that of the isolated eIF4Es. This suggests that, although interaction with VPg directly affects the degree of flexibility, it does not significantly influence the degree of compaction of eIF4E. The electrostatic profiles of eIF4E and VPg proteins revealed predominantly cationic and anionic charges, respectively. The distribution of positive charges in the 5’ cap recognition region of mRNA by eIF4E is critical for efficient translation initiation in eukaryotes. Furthermore, this same characteristic allows the recognition of VPg, which has a negative charge, resembling the physicochemical nature of N7-methylguanosine (m7G) of the host’s mRNA 5’cap region [13]. This mechanism is shared with other viruses of the Potyviridae family, known for hijacking the host’s translation machinery to ensure successful infection.

The findings revealed by the analysis of the eIF4E-VPg-binding interface reveal that its stability does not depend on a single force but on a network of intramolecular interactions, combining both hydrophobic and electrostatic effects. Docking analysis and binding free energy calculations revealed distinct thermodynamic profiles among the cultivars. The Boca Negra cultivar, for instance, exhibited highly thermodynamically favorable binding interactions, whereas BRS Cauamé displayed a less favorable energetic profile. Suggestive divergent binding profiles were also observed, such as the use of the polar residue Arg-120 by BRS Xiquexique and the hydrophobic residue Pro-73 by the Bajão cultivar. Hydrophobic residues like Trp-68 and Ile-74 were prominent, and electrostatic forces involving charged residues appear to be crucial for both the orientation and energetic stabilization of the complex.

Furthermore, one of the most significant findings was the identification of interaction *“hotspots”*, both conserved and cultivar-specific patterns. The residue Arg-118 emerged as a fundamental and convergent anchoring point in four (Boca Negra, BRS Cauamé, BRS Xiquexique, and IT85F-2687) of the five cultivars, while specific residues, such as Gln-121 in BRS Cauamé and the Pro-73 and Ile-74 pair in Bajão, likely determine the differences in binding affinity. These variations in the interface may be the molecular basis for the observed differences in susceptibility to viral infection. Although the data is computational, it provides a solid basis for prioritizing these residues in future site-directed mutagenesis studies, with experimental validation being a critical next step for developing durable resistance strategies.

The physicochemical conservation of VPg enables it to mimic the 5’cap structure of eukaryotic mRNA. This feature in viruses of the Potyviridae family may be one of the keys to their high infection potential across diverse plant hosts, including potato, tomato, passion fruit, and peanut [17]. In vitro and NMR-based structural studies have demonstrated that PVY VPg can interact with human eIF4E, reflecting the strong conservation of the primary sequence and structural folding of eIF4E across eukaryotes [13]. This conservation of physicochemical nature in both VPg and eukaryotic eIF4E may be one of the factors explaining the broad infection capacity of Potyviridae family viruses in a wide variety of vegetables belonging to different genera and families.

Finally, our analysis of the eIF4E-VPg interaction suggests that additional factors, independently of eIF4E, may influence the process of susceptibility or resistance to CABMV in *V. unguiculata* cultivars. The results also indicate that resistance to viral infection in *V. unguiculata* is not absolute. Therefore, further investigations are essential to elucidate other additional resistance mechanisms against Potyviruses.

## 5. Conclusions

The results obtained suggest that mutations in the *eIF4E* gene affect the structural properties of the protein and, consequently, its interaction with the CABMV VPg protein, potentially modulating the resistance or susceptibility of *V. unguiculata*. The mutation data presented in this study suggests that they may be associated with the resistance or susceptibility of *V. unguiculata* cultivars to CABMV, as confirmed with bioassays, where most cultivars harboring susceptibility-associated mutations exhibited symptoms. In turn, some of the cultivars with resistance-associated mutations remained asymptomatic. Supporting the experimental findings, a suite of computational analyses, including MD simulations, docking, and binding free energy calculations, revealed that structural adjustments both within the eIF4E protein and at the eIF4E-VPg interface dictate binding affinity. The stability of this interaction is critically dependent not only on hydrophobic and electrostatic forces but also on a more complex network of interactions required to maintain the bond. Also, the interface and energetic analyses pinpointed a set of key residues driving this interaction, which represent promising targets for enabling a basis for a deeper future understanding.

These findings reinforce the role of eIF4E in the response to CABMV and provide a basis for selecting genotypes resistant to CABMV and other plant viruses dependent on translation factors. Furthermore, this study presents, for the first time, approaches involving protein modeling, MD simulations, and docking analysis, providing strong evidence of the association between specific mutations and susceptibility/resistance of *V. unguiculata* cultivars to agriculturally important Potyviruses. The results also highlight the complexity of the mechanisms involved in the virus–host interaction, suggesting that additional, yet unidentified, factors may be indirectly acting on the condition of susceptibility/resistance to CABMV. Our data reinforces the importance of the *eIF4E* gene as a potential target in generating CABMV-resistant cultivars via gene editing.

## 6. Future Perspectives

The data obtained in this study indicates some key regions and mutations of the *eIF4E* gene associated with cowpea resistance to CABMV. As future perspectives, the target regions may be promising for gene editing approaches, such as CRISPR/Cas9. Furthermore, the identified polymorphisms are valuable for application in marker-assisted selection (MAS) in breeding programs, significantly reducing the time required for the identification and introgression of resistance genes in cultivars of interest.

## Figures and Tables

**Table 1:** Mutations identified in the *eIF4E* gene sequence and the corresponding amino acid sequences of proteins from three cowpea cultivars: Boca Negra, BR14 Mulato (susceptible to CABMV), and IT85F-2687 (resistant to CABMV). Of the five mutations detected, three (*) differentiate the susceptible cultivars from the resistant one.

POSITION	TYPE	CULTIVAR/Condition		
		Susceptible		Resistant
		BOCA NEGRA	BR14 MULATO	IT85F-2687
203	Nucleotide	Cytosine (C)	Cytosine (C)	Guanine (G) *
224	Nucleotide	Cytosine (C)	Adenine (A)	Adenine (A)
325	Nucleotide	Guanine (G)	Guanine (G)	Cytosine (C) *
329	Nucleotide	Cytosine (C)	Cytosine (C)	Thymine (T) *
520	Nucleotide	Adenine (A)	Thymine (T)	Adenine (A)
68	Amino acid	Proline (Pro)	Proline (Pro)	Arginine (Arg) *
75	Amino acid	Alanine (Ala)	Aspartate (Asp)	Aspartate (Asp)
109	Amino acid	Glycine (Gly)	Glycine (Gly)	Arginine (Arg) *
110	Amino acid	Alanine (Ala)	Alanine (Ala)	Valine (Val) *
174	Amino acid	Tyrosine (Tyr)	Asparagine (Asn)	Asparagine (Asn)

**Table 2:** Analysis of the 27 cowpea cultivars for their response to CABMV infection (resistant/susceptible), based on PCR results and bioassays, compared with published reports. *: negative PCR (no amplification); **: no published data available regarding the cultivar’s response to CABMV.

GENOTYPES	THIS STUDY		LITERATURE DATA	
	PCR	Bioassay	Reaction	Reference
1—Santo Inácio	Susceptible	Susceptible	**	
2—Pingo de Ouro	Susceptible	Susceptible	Susceptible	[37]
3—BR 14 Mulato	Susceptible	Susceptible	Susceptible	[47,48]
4—BRS Xiquexique	Susceptible	Susceptible	Susceptible	[48]
5—BRS Tumucumaque	Resistant	Susceptible	Susceptible	[48]
6—Inhuma	Susceptible	Susceptible	**	
7—Boca Negra	Susceptible	Susceptible	**	
8—João Paulo II	Susceptible	Susceptible	**	
9—IT85F-2687	Resistant	Resistant	Resistant	[48]
10—BR 1 Poty	Susceptible	Susceptible	Susceptible	[37]
11—BRS Maratoã	Susceptible	Susceptible	Susceptible/ Resistant	[37,47]
12—BRS Cauamé	Resistant	Susceptible	Susceptible/Resistant	[45,46]
13—BRS Guariba	Resistant	Resistant	Resistant/Susceptible	[47,48,49]
14—BRS Itaim	Susceptible	Susceptible	Resistant	[47]
15—BRS Paraguaçu	Susceptible	Susceptible	**	
16—L.950.002	Resistant	Resistant	**	
17—Miranda IPA 207	Susceptible	Resistant	**	
18—IPA 206	Resistant	Resistant	**	
19—BRS Juruá	Susceptible	Susceptible	Susceptible/Resistant	[36,47]
20—Canapu	Susceptible	Susceptible	**	
21—BR10 Piauí	Susceptible	Susceptible	Susceptible	[48]
22—Corujinha	Resistant	Susceptible	**	
23—TVU-966	*	Resistant	Resistant	[47,50]
24—Manteguinha Santarém	Susceptible	Resistant	Resistant	[48]
25—IT81D-1053	Resistant	Resistant	Resistant	[48]
26—Sempre Verde Salgueiro	Susceptible	Susceptible	Susceptible	[50]
27—Bajão	*	Resistant	Resistant	[48]

**Table 3:** Data on HADDOCK score, Gibbs free energy difference (Theorical DDG), and interaction area related to VPg-eIF4E complex formation.

COMPLEXES	HADDOCK		ROSETTA		HAWKDOCK MM-GBSA
	Score	Interaction Area (Å2)	Theorical DDG (REUs)	Molecular Surface (Å2)	Predicted Binding Free Energy of Complex (kcal/mol)
eIF4E (Bajão)/VPg	−75.9	1195.7	−35.724	632.566	−84.23
eIF4E (Boca Negra)/VPg	−91.2	1424.1	−41.459	684.034	−98.21
eIF4E (BRS Cauamé)/VPg	−72.3	1316.4	−20.840	455.246	−68.52
eIF4E (BRS Xiquexique)/VPg	−102.9	1944.6	−23.612	453.629	−72.97
eIF4E (IT85F-2687)/VPg	−115.5	1820.6	−30.910	518.889	−82.22

## Data Availability

Data is contained within the article or Supplementary Materials, and further inquiries can be directed to the corresponding authors.

## Supplementary Materials

The following are available online at https://www.mdpi.com/article/10.3390/v17081050/s1, Figure S1: Conserved domain of VPg proteins from different plant viruses. Figure S2: Agarose gel electrophoresis (1.5%) of eIF4E gene CDS amplifications from 27 cowpea cultivars. Figure S3: Percent identity matrix of eIF4E gene sequences from 27 V. unguiculata cultivars, performed in MEGA (v. 11). Figure S4: Alignment between cowpea cultivars. The three reference sequences (with the Full tag), with the 27 cowpea cultivars (1 to 27). Figure S5: Alignment of eIF4E proteins from cowpea cultivars that showed characteristic mutations. Figure S6: Alignment of the three-dimensional structures of V. unguiculata eIF4E proteins. Figure S7: Graphs referring to the molecular dynamics of CABMV VPg. Figure S8: Annotated RMSF plot highlighting key flexibility peaks in eIF4E.Figure S9: Graphical representation of b-factor values in eIF4E protein models, highlighting flexibility peaks that vary to warmer (red/orange) and thicker tones. Table S1. Per-residue binding free energy decomposition for eIF4E variants from different cultivars interacting with CABMV VPg. Table S2: Intermolecular contacts at the eIF4E-VPg interface for the Bajão cultivar. Table S3: Intermolecular contacts at the eIF4E-VPg interface for the Boca Negra cultivar. Table S4: Intermolecular contacts at the eIF4E-VPg interface for the BRS Cauamé cultivar. Table S5: Intermolecular contacts at the eIF4E-VPg interface for the BRS Xiquexique cultivar. Table S6: Intermolecular contacts at the eIF4E-VPg interface for the BRS IT85F-2687 cultivar. Table S7: Summary of the most frequent eIF4E residues at the interaction interface with VPg. Table S8: Hydrogen bond occupancy (%) at the eIF4E–VPg interface for the Bajão cultivar. Table S9: Hydrogen bond occupancy (%) at the eIF4E–VPg interface for the Boca Negra cultivar. Table S10: Hydrogen bond occupancy (%) at the eIF4E–VPg interface for the BRS Cauamé cultivar. Table S11: Hydrogen bond occupancy (%) at the eIF4E–VPg interface for the BRS Xiquexique cultivar. Table S12: Hydrogen bond occupancy (%) at the eIF4E–VPg interface for the IT85F-2687 cultivar.

## Abbreviations

+ssRNA	Positive-sense single-stranded RNA
A	Adenine
Ala	Alanine
Asn	Asparagine
Asp	Aspartate
C	Cytosine
CABMV	*Cowpea aphid-borne mosaic virus*
cDNA	Complementary DNA
CDS	Coding sequence
CPSMV	*Cowpea severe mosaic virus*
DAIs	Days after inoculation
DDG	Delta Delta G
eIF4E	Eukaryotic translation initiation factor 4E
F	Forward
G	Guanine
Gln	Glutamine
Glu	Glutamate
Gly	Glycine
HBs	Hydrogen bonds
m7GpppN	7-Methylguanosine
MD	Molecular dynamics
mRNA	Messenger RNA
NMR	Nuclear magnetic resonance
PAE	Predicted Aligned Error
PDB	Protein Data Bank
pLDDT	Predicted Local Distance Difference Test
Poly-A	Polyadenylated tail
Pro	Proline
PSE	Electrostatic Surface Potential
PVMV	*Pepper veinal mottle virus*
PVY	*Potato virus Y*
R	Reverse
*R* genes	Resistance genes
REU	Rosetta Energy Unit
RG	Radius of gyration
RMSD	Root Mean Square Deviation
RMSF	Root Mean Square Fluctuation
*S* genes	Susceptibility genes
SNPs	Single-nucleotide polymorphisms
T	Thymine
Trp	Tryptophan
Tyr	Tyrosine
UMP	Uridine monophosphate
Val	Valine
VPg	Viral protein genome-linked
