# Supplementary material for: Deciphering Cowpea Resistance to Potyvirus: Assessment of eIF4E Gene Mutations and Their Impact on the eIF4E-VPg Protein Interaction
Source: Viruses. 2025 Jul 28;17(8):1050. doi: 10.3390/v17081050 (PMC12390716; doi:10.3390/v17081050)
Supplement: Supplementary file 1 [file viruses-17-01050-s001.zip › supplementary_images.pdf]

Supplementary Materials

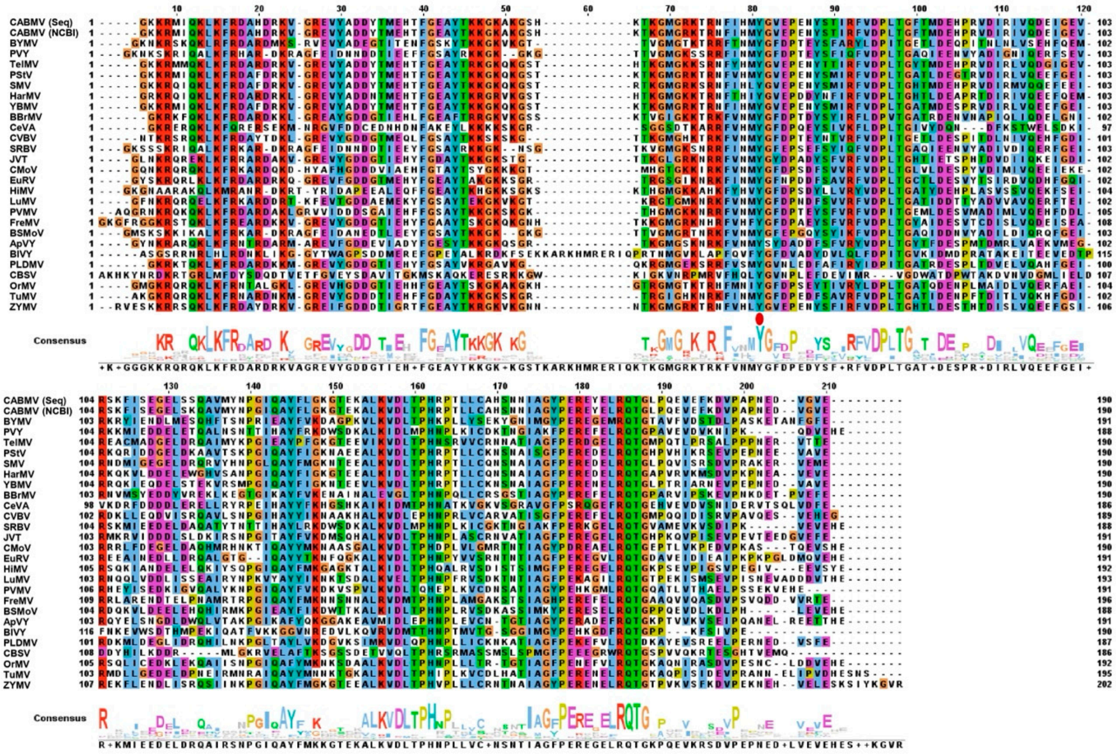

Figure S1: Conserved domain of VPg proteins from different plant viruses. *Cowpea aphid-borne mosaic virus* (CABMV Seq), *Cowpea aphid-borne mosaic virus* (CABMV NCBI), *Bean yellow mosaic virus* (BYMV), *Potato virus Y* (PVY), *Telosma mosaic virus* (TelMV), *Peanut stripe virus* (PStV), *Soybean mosaic virus* (SMV), *Hardenbergia mosaic virus* (HarMV), *Yambean mosaic virus* (YBMV), *Banana bract mosaic virus* (BBrMV), *Cyrtanthus elatus virus A* (CeVA), *Cucurbit vein banding virus* (CVBV), *Sunflower ring blotch virus* (SRBV), *Jasmine virus T* (JVT), *Callistephus mottle virus* (CMoV), *Euphorbia ringspot virus* (EuRV), *Hippeastrum mosaic virus* (HiMV), *Lupinus mosaic virus* (LuMV), *Pepper veinal mottle virus* (PVMV), *Freesia mosaic virus* (FreMV), *Brugmansia suaveolens mottle virus* (BSMoV), *Apium virus Y* (ApVY), *Blackberry virus Y* (BlVY), *Papaya leaf distortion mosaic virus* (PLDMV), *Cassava brown streak virus* (CBSV), *Ornithogalum mosaic virus* (OrMV), *Turnip mosaic virus* (TuMV), *Zucchini yellow mosaic virus* (ZYMV).

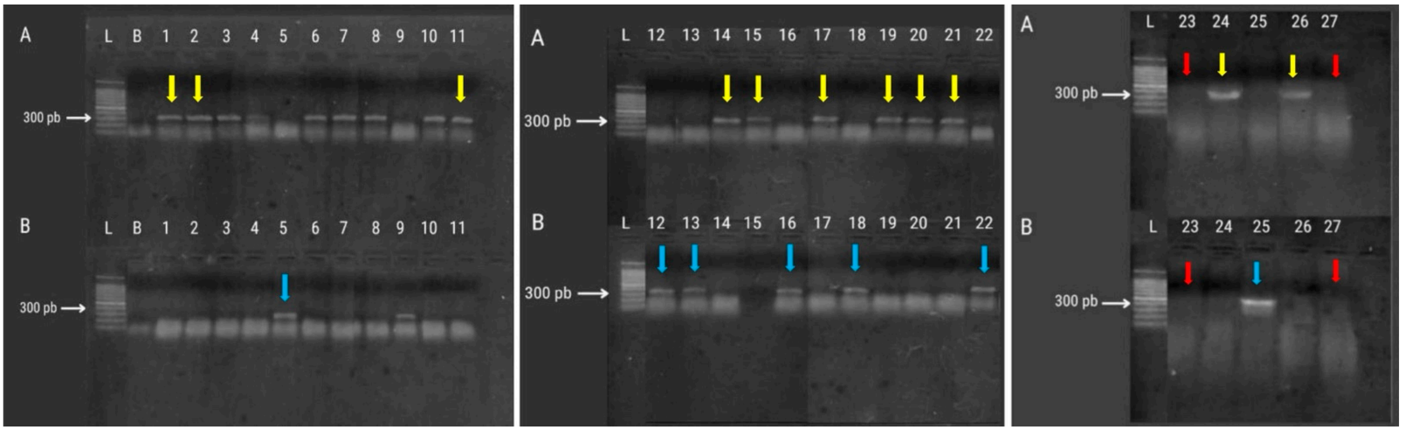

Figure S2: Agarose gel electrophoresis (1.5%) of *eIF4E* gene CDS amplifications from 27 cowpea cultivars. (A) fragments amplified with primer amplifying the mutation indicative of susceptibility; (B) fragments amplified with primer

amplifying the mutation indicative of resistance to CABMV. Cultivars numbered 23 and 27 did not amplify with any of the primers.

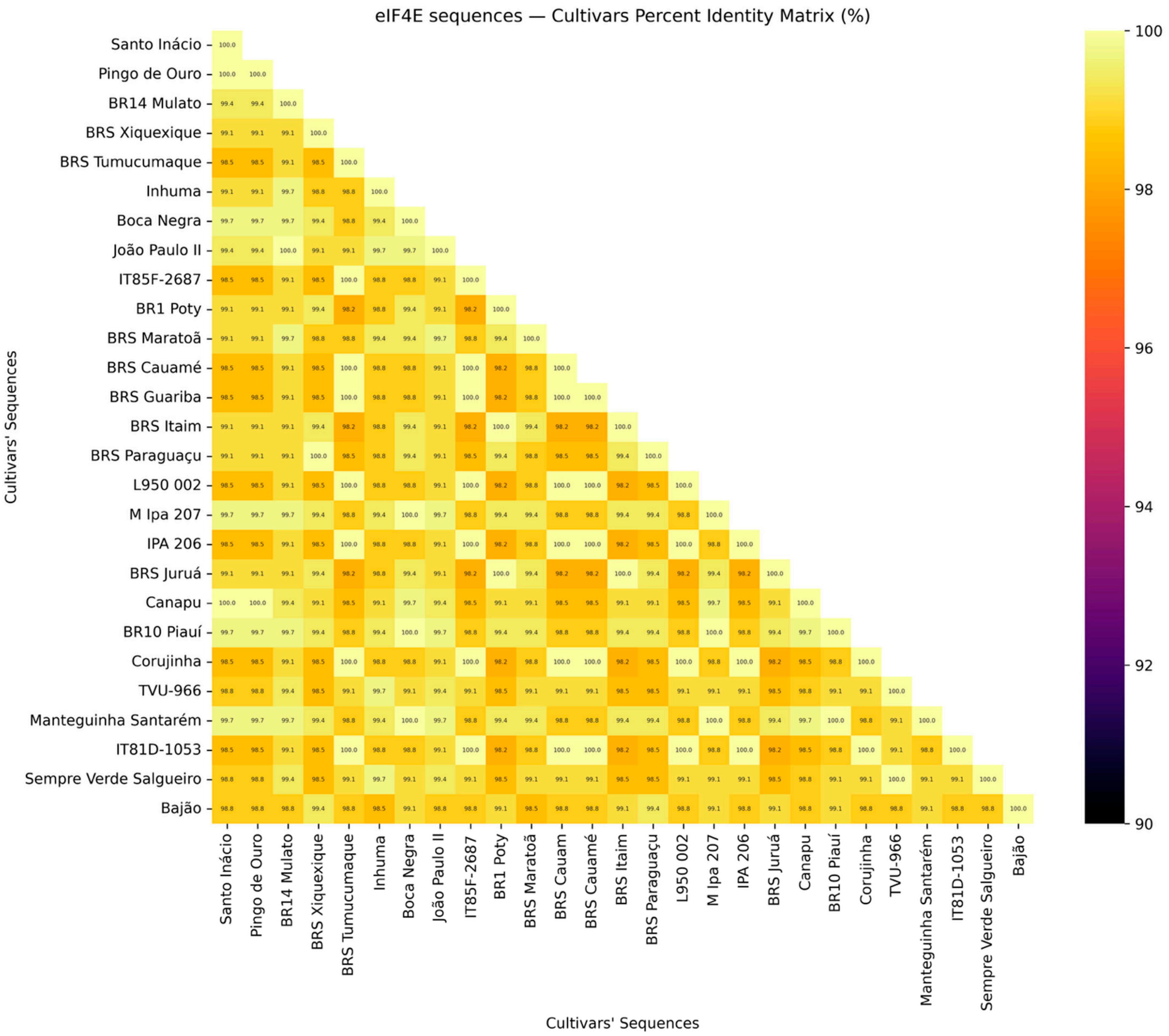

Figure S3: Percent identity matrix of eIF4E gene sequences from 27 *V. unguiculata* cultivars, performed in MEGA (v. 11). Conservation among cowpea cultivars above 98%.

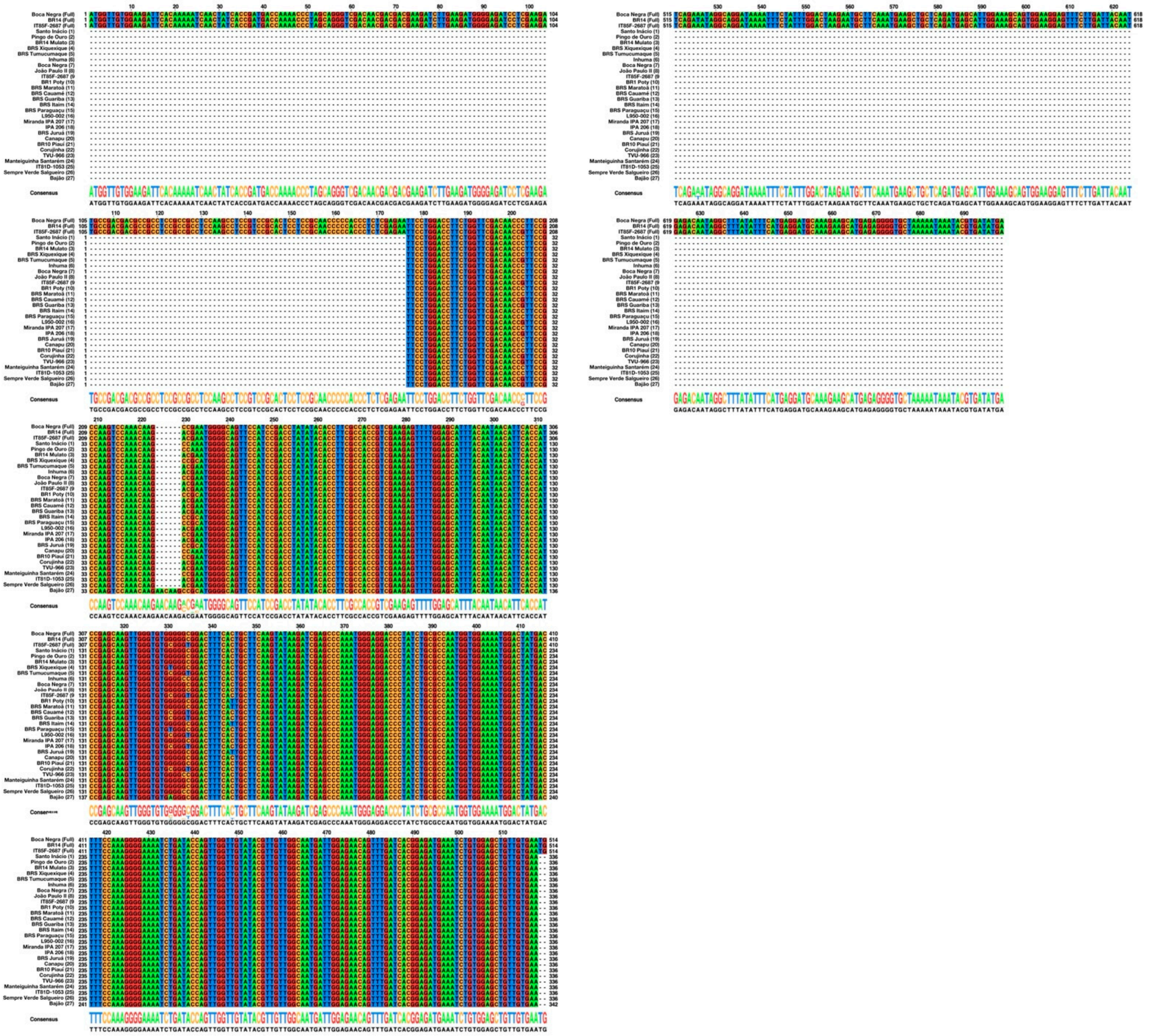

Figure S4: Alignment between cowpea cultivars. The three reference sequences (with the Full tag), with the 27 cowpea cultivars (1 to 27). Red dot indicates the C/G mutation at position 203; black dot indicates the 6 bp insertion in the Bajão cultivar; green dot indicates mutations at position 325 among cowpea cultivars.

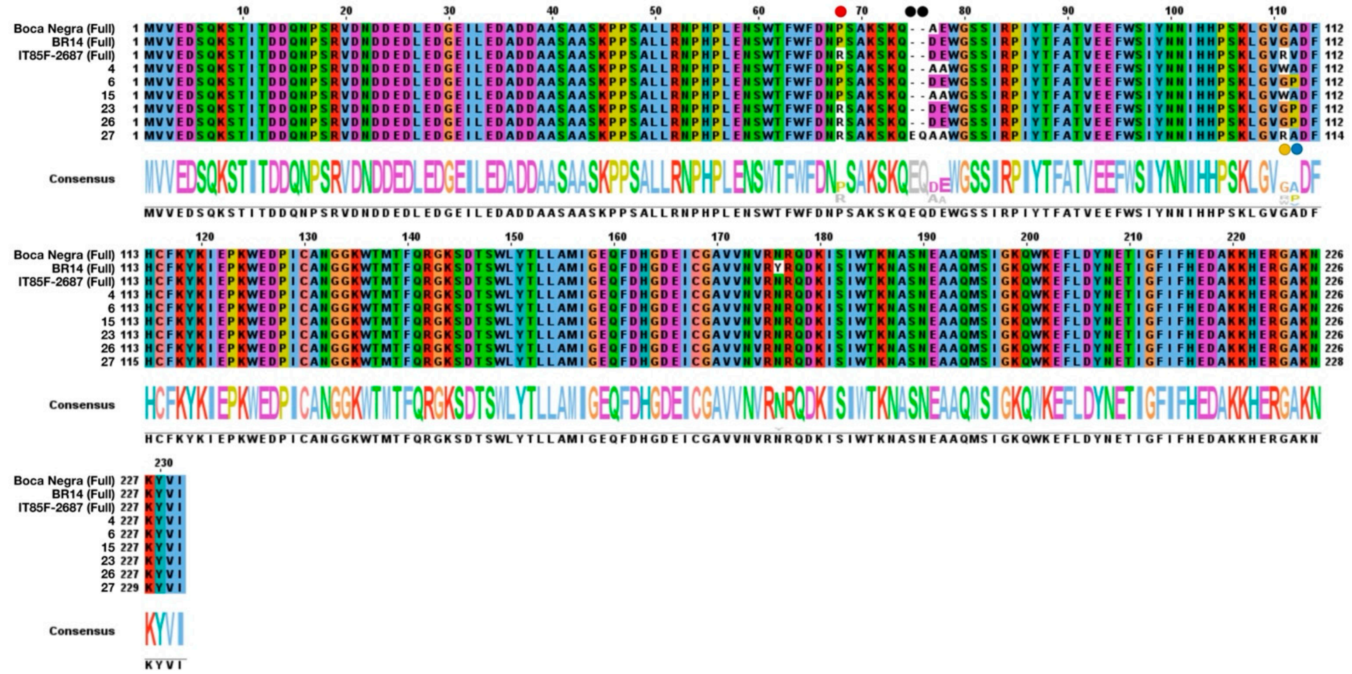

Figure S5: Alignment of eIF4E proteins from cowpea cultivars that showed characteristic mutations. At position 68 the substitutions are non-synonymous (red filled circle). Positions 76 and 77, addition of two amino acid residues (Glu: glutamate, Gln: glutamine) in cultivar 27 - Bajão (black filled circle). Cultivars 4 - BRS Xiquexique and 15 - BRS Paraguaçu show a synonymous substitution Trp111Gly, in relation to the other possibly susceptible cultivars (yellow filled circle). Cultivars 6 - Inhuma, 23 - TVU-966 and 26 - Sempre Verde Salgueiro, have a substitution at position 111 (Pro), differentiating them from the other cultivars (blue filled circle). Highlighted in yellow is the amino acid sequence of the three cultivars used as reference.

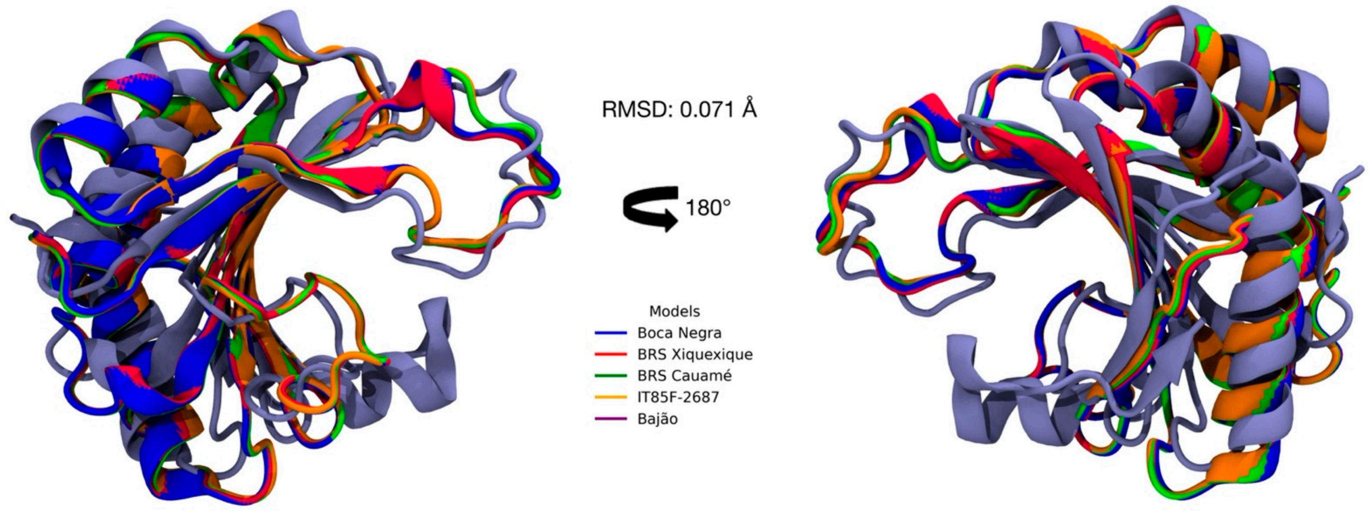

Figure S6: Alignment of the three-dimensional structures of *V. unguiculata* eIF4E proteins. RMSD of 0.071 Å (PYMOL v. 3.1).

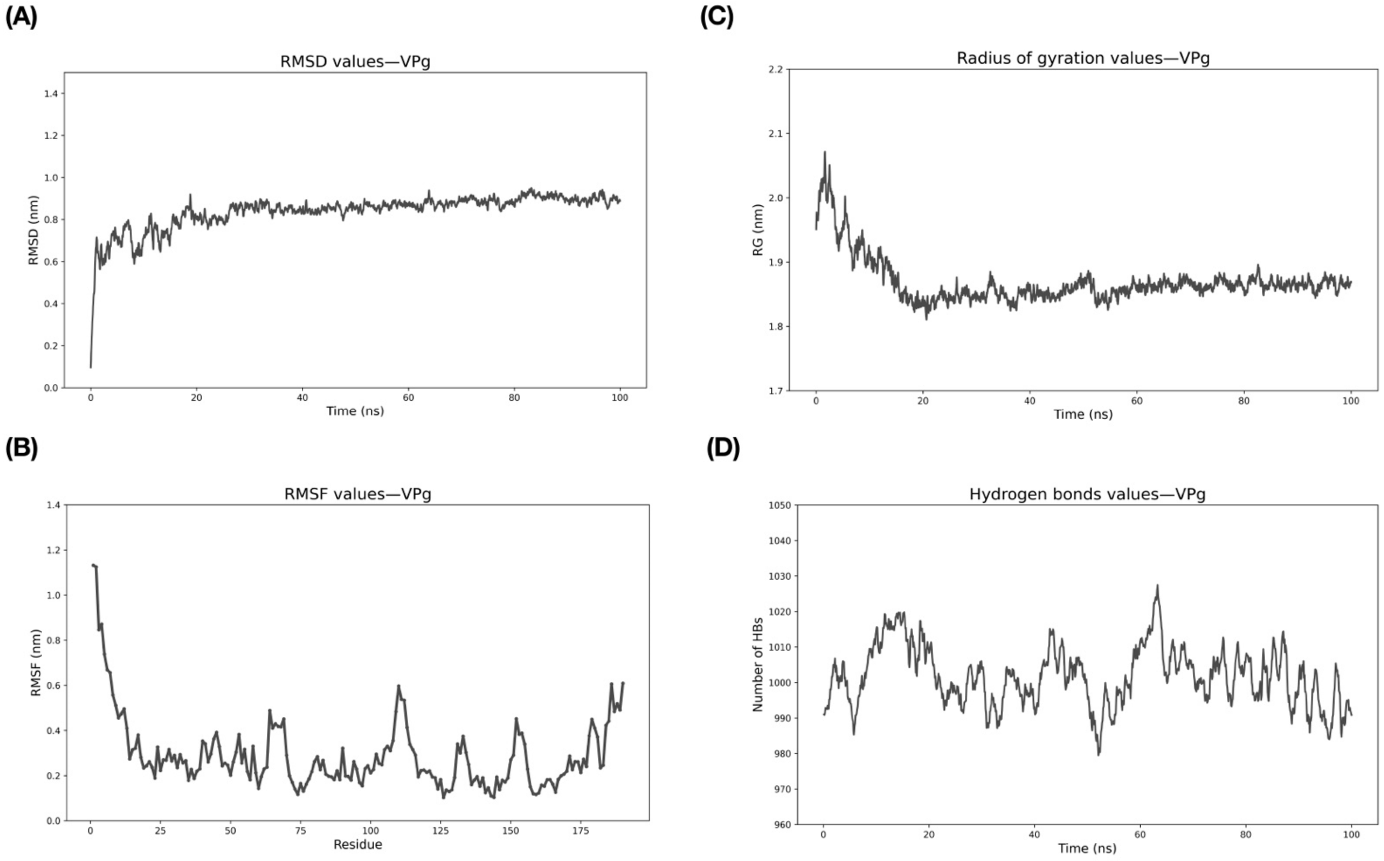

Figure S7: Graphs referring to the molecular dynamics of CABMV VPg. (A) RMSD values - VPg, (B) RMSF values - VPg, (C) Radius of Gyration values - VPg, (D) Hydrogen Bonds values - VPg.

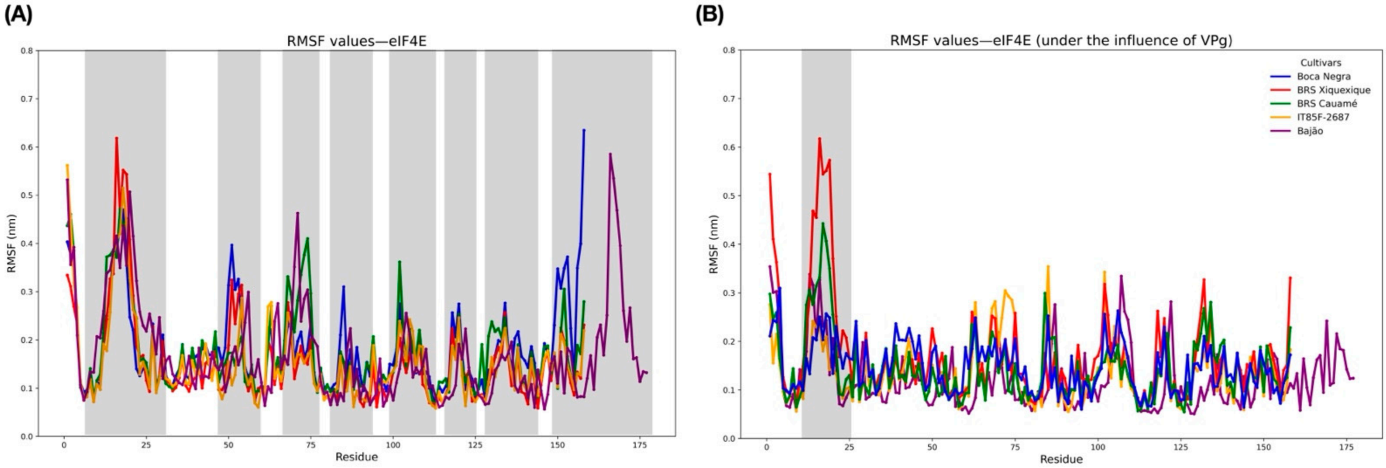

Figure S8: Annotated RMSF plot highlighting key flexibility peaks in eIF4E. This figure presents the same data as Figure 5, but with translucent gray rectangles added to emphasize regions of high conformational flexibility that are discussed in the text, such as the N-terminal domain. (A) Monomeric eIF4E proteins. (B) eIF4E proteins in complex with VPg.

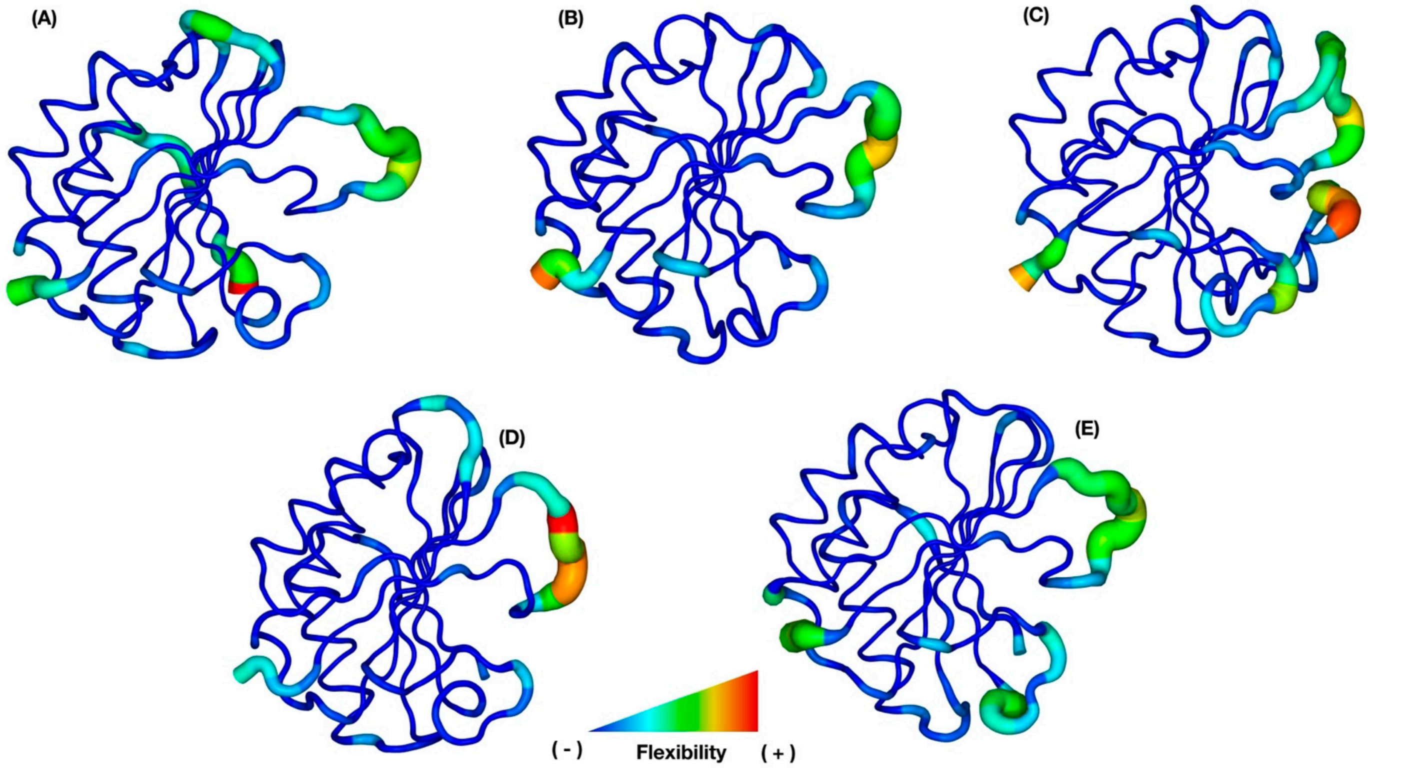

Figure S9: Graphical representation of b-factor values in eIF4E protein models, highlighting flexibility peaks that vary to warmer (red/orange) and thicker tones. Cultivars: (A) Boca Negra, (B) IT85F-2687, (C) Bajão, (D) BRS Xiquexique, (E) BRS Cauamé. Flexibility ranging from -1 (less flexible) to +1 (more flexible).
