# Supplementary material for: Deciphering Cowpea Resistance to Potyvirus: Assessment of eIF4E Gene Mutations and Their Impact on the eIF4E-VPg Protein Interaction
Source: Viruses. 2025 Jul 28;17(8):1050. doi: 10.3390/v17081050 (PMC12390716; doi:10.3390/v17081050)
Supplement: Supplementary file 1 [file viruses-17-01050-s001.zip › supplementary_tables.pdf]

Table S1. Per-residue binding free energy decomposition for eIF4E variants from different cultivars interacting with CABMV VPg. The table lists the top 10 amino acid residues with the most significant contributions to the binding free energy (ΔGbind), as calculated by the MM/GBSA method from the HawkDock server. The analysis was performed for each of the five eIF4E-VPg complexes, and all energy values are reported in kcal/mol.

Energy Rank	eIF4E (Bajão)		eIF4E (Boca Negra)		eIF4E (BRS Cauamé)		eIF4E (BRS Xiquexique)		eIF4E (IT85F-2687)	
	Residue	ΔGbind (kcal/mol)	Residue	ΔGbind (kcal/mol)	Residue	ΔGbind (kcal/mol)	Residue	ΔGbind (kcal/mol)	Residue	ΔGbind (kcal/mol)
**#1**	Pro-73	-5,65	Trp-68	-7,33	Gln-121	-6,07	Arg-120	-14,89	Gln-19	-7,75
**#2**	Ile-74	-4,53	Arg-118	-6,96	Ile-157	-4,47	Arg-118	-6,77	Arg-120	-5,72
**#3**	His-108	-4,15	Pro-71	-4,59	Arg-120	-4,45	Asn-151	-5,25	Arg-54	-5,15
**#4**	Arg-29	-3,33	Lys-16	-4,5	Trp-68	-4,15	Thr-153	-4,53	Lys-16	-4,42
**#5**	Tyr-64	-3,12	Asn-119	-3,87	Gln-84	-3,56	Asn-119	-4,03	Ala-15	-3,92
**#6**	Asn-132	-2,87	Asp-70	-3,12	Thr-82	-3,23	Leu-148	-2,31	Arg-118	-3,87
**#7**	Asn-135	-2,48	Trp-22	-3,08	Lys-123	-2,37	Gln-121	-1,91	Gln-121	-2,98
**#8**	Trp-70	-2,28	Ala-20	-2,91	Arg-118	-2,33	Glu-146	-1,71	Phe-156	-2,92
**#9**	Glu-111	-2,11	Asn-75	-2,41	Gly-155	-2,25	Thr-82	-1,59	Phe-158	-2,42
**#10**	Glu-3	-1,67	Asn-116	-2,39	Asn-75	-1,79	Ile-157	-1,4	Asn-133	-2,35

Table S2: Intermolecular contacts at the eIF4E-VPg interface for the Bajão cultivar. The table lists the specific non-covalent interactions between eIF4E (Chain A) and VPg (Chain B), identified from the most representative complex structure. The analysis includes all eIF4E residues located within a 5 Å radius of VPg. For each interacting pair, the table specifies the residues involved, the minimum distance between them in angstroms (Å), and the categorized interaction type (e.g., Hydrogen Bond, Salt Bridge, Hydrophobic, or van der Waals).

eIF4E	Residue 1	VPg	Residue 2	Minimum Distance (Å)	Interaction Type
**A**	GLU3	B	LYS45	3.89	Salt Bridge
**A**	SER5	B	LYS45	3.95	van der Waals Contact
**A**	ARG13	B	SER105	3.04	van der Waals Contact
**A**	ARG13	B	LYS106	2.62	Hydrogen Bond
**A**	ARG13	B	SER109	3.38	van der Waals Contact

**A**	ARG13	B	ILE108	4.43	van der Waals Contact
**A**	ARG13	B	PHE107	4.45	van der Waals Contact
**A**	SER14	B	LEU113	4.57	van der Waals Contact
**A**	TRP24	B	LEU113	3.74	Hydrophobic Interaction
**A**	GLY25	B	LEU113	4.01	van der Waals Contact
**A**	GLY25	B	SER115	2.16	Hydrogen Bond
**A**	GLY25	B	SER114	3.80	van der Waals Contact
**A**	SER26	B	LEU113	3.81	van der Waals Contact
**A**	SER26	B	SER115	3.50	van der Waals Contact
**A**	SER26	B	MET119	3.99	van der Waals Contact
**A**	SER26	B	SER114	4.99	van der Waals Contact
**A**	SER26	B	SER105	4.91	van der Waals Contact
**A**	SER26	B	SER109	4.07	van der Waals Contact
**A**	SER26	B	ILE108	4.79	van der Waals Contact
**A**	SER27	B	SER115	4.47	van der Waals Contact
**A**	SER27	B	SER105	3.89	van der Waals Contact
**A**	ILE28	B	SER115	3.96	van der Waals Contact
**A**	ARG29	B	GLU102	3.96	Salt Bridge
**A**	TYR64	B	LYS40	3.91	van der Waals Contact
**A**	TYR64	B	LYS41	4.28	van der Waals Contact
**A**	TYR64	B	ALA44	3.06	van der Waals Contact
**A**	TYR64	B	LYS45	3.88	van der Waals Contact
**A**	LYS65	B	LYS40	2.02	Hydrogen Bond

**A**	LYS65	B	GLN116	4.13	van der Waals Contact
**A**	LYS65	B	GLU163	4.30	van der Waals Contact
**A**	ILE66	B	LYS40	3.84	van der Waals Contact
**A**	GLU67	B	LYS40	4.46	van der Waals Contact
**A**	GLU67	B	GLN116	1.83	Hydrogen Bond
**A**	GLU67	B	SER114	1.72	Hydrogen Bond
**A**	GLU67	B	SER115	3.27	Hydrogen Bond
**A**	GLU67	B	ALA117	2.04	Hydrogen Bond
**A**	GLU67	B	VAL118	4.19	van der Waals Contact
**A**	PRO68	B	LEU113	4.35	Hydrophobic Interaction
**A**	LYS69	B	LEU113	3.17	van der Waals Contact
**A**	LYS69	B	SER114	1.94	Hydrogen Bond
**A**	LYS69	B	TYR24	1.84	Hydrogen Bond
**A**	LYS69	B	GLU112	2.52	Salt Bridge
**A**	LYS69	B	ARG12	3.58	van der Waals Contact
**A**	LYS69	B	ALA117	4.01	van der Waals Contact
**A**	TRP70	B	GLU112	3.24	van der Waals Contact
**A**	TRP70	B	GLY111	2.45	van der Waals Contact
**A**	TRP70	B	LEU113	2.46	Hydrophobic Interaction
**A**	GLU71	B	LYS10	3.16	Salt Bridge
**A**	GLU71	B	TYR24	4.29	van der Waals Contact
**A**	ASP72	B	TYR24	2.47	van der Waals Contact
**A**	PRO73	B	TYR24	3.13	van der Waals Contact

**A**	PRO73	B	ASP26	3.42	van der Waals Contact
**A**	PRO73	B	LYS8	3.52	van der Waals Contact
**A**	PRO73	B	ALA25	4.59	Hydrophobic Interaction
**A**	PRO73	B	LYS10	3.99	van der Waals Contact
**A**	PRO73	B	LEU9	4.65	Hydrophobic Interaction
**A**	ILE74	B	ASP26	3.87	van der Waals Contact
**A**	ILE74	B	TYR24	3.78	van der Waals Contact
**A**	ILE74	B	ALA25	3.05	Hydrophobic Interaction
**A**	ILE74	B	LYS40	4.23	van der Waals Contact
**A**	ILE74	B	ASP27	4.42	van der Waals Contact
**A**	ILE74	B	VAL23	4.48	Hydrophobic Interaction
**A**	ASN77	B	LYS8	4.05	van der Waals Contact
**A**	ASP107	B	LYS2	3.70	Salt Bridge
**A**	HIS108	B	LYS2	1.87	van der Waals Contact
**A**	HIS108	B	ARG4	1.88	Hydrogen Bond
**A**	HIS108	B	MET5	4.26	van der Waals Contact
**A**	HIS108	B	LYS3	3.35	van der Waals Contact
**A**	GLY109	B	TYR28	2.86	van der Waals Contact
**A**	GLY109	B	LYS2	3.88	van der Waals Contact
**A**	GLY109	B	MET5	3.06	van der Waals Contact
**A**	GLY109	B	ARG4	4.02	van der Waals Contact
**A**	GLY109	B	LYS3	4.37	van der Waals Contact
**A**	ASP110	B	TYR28	2.73	van der Waals Contact

**A**	ASP110	B	MET5	3.67	van der Waals Contact
**A**	ASP110	B	LYS41	4.84	van der Waals Contact
**A**	ASP110	B	ASP27	4.98	van der Waals Contact
**A**	ASP110	B	LYS8	4.40	van der Waals Contact
**A**	GLU111	B	MET5	3.93	van der Waals Contact
**A**	GLU111	B	ARG4	4.48	van der Waals Contact
**A**	LYS131	B	LYS8	3.92	van der Waals Contact
**A**	LYS131	B	ASP26	3.16	Salt Bridge
**A**	LYS131	B	ASP27	4.32	van der Waals Contact
**A**	LYS131	B	TYR28	4.76	van der Waals Contact
**A**	ASN132	B	LYS8	2.24	Hydrogen Bond
**A**	ASN132	B	MET5	1.91	van der Waals Contact
**A**	ASN132	B	ILE6	2.72	van der Waals Contact
**A**	ASN132	B	ARG4	4.11	van der Waals Contact
**A**	ALA133	B	ILE6	4.25	Hydrophobic Interaction
**A**	SER134	B	ILE6	3.31	van der Waals Contact
**A**	ASN135	B	ARG4	2.03	van der Waals Contact
**A**	ASN135	B	MET5	4.04	van der Waals Contact
**A**	ASN135	B	ILE6	4.89	van der Waals Contact
**A**	ALA138	B	ARG4	4.37	van der Waals Contact

Table S3: Intermolecular contacts at the eIF4E-VPg interface for the Boca Negra cultivar. The table lists the specific non-covalent interactions between eIF4E (Chain A) and VPg (Chain B), identified from the most representative complex structure. The analysis includes all eIF4E residues located within a 5 Å radius of VPg. For

each interacting pair, the table specifies the residues involved, the minimum distance between them in angstroms (Å), and the categorized interaction type (e.g., Hydrogen Bond, Salt Bridge, Hydrophobic, or van der Waals).

eIF4E	Residue 1	VPg	Residue 2	Minimum Distance (Å)	Interaction Type
A	SER14	B	LEU113	3.61	van der Waals Contact
A	ALA15	B	LEU113	3.55	Hydrophobic Interaction
A	ALA15	B	ILE108	4.73	Hydrophobic Interaction
A	ALA15	B	SER105	4.12	van der Waals Contact
A	ALA15	B	GLU190	4.70	van der Waals Contact
A	LYS16	B	SER105	3.49	van der Waals Contact
A	LYS16	B	GLU190	2.70	Salt Bridge
A	LYS16	B	SER115	4.64	van der Waals Contact
A	LYS16	B	MET119	4.91	van der Waals Contact
A	SER17	B	SER105	2.19	Hydrogen Bond
A	SER17	B	SER115	1.57	Hydrogen Bond
A	SER17	B	MET119	4.21	van der Waals Contact
A	SER17	B	TYR120	3.11	van der Waals Contact
A	SER17	B	GLY101	3.97	van der Waals Contact
A	SER17	B	GLU102	4.09	van der Waals Contact
A	SER17	B	ASP98	4.57	van der Waals Contact
A	LYS18	B	SER115	3.25	van der Waals Contact
A	LYS18	B	TYR120	1.85	Hydrogen Bond
A	LYS18	B	ASP98	3.41	Salt Bridge
A	GLN19	B	SER115	4.09	van der Waals Contact

A	ALA20	B	SER115	3.41	van der Waals Contact
A	ALA20	B	SER114	3.57	van der Waals Contact
A	ALA20	B	LEU113	3.10	Hydrophobic Interaction
A	ALA20	B	MET119	3.97	Hydrophobic Interaction
A	GLU21	B	LEU113	3.57	van der Waals Contact
A	TRP22	B	LEU113	3.49	Hydrophobic Interaction
A	TRP22	B	GLY111	3.14	van der Waals Contact
A	TRP22	B	SER109	4.18	van der Waals Contact
A	ASP56	B	GLY111	4.87	van der Waals Contact
A	ASP56	B	SER109	4.93	van der Waals Contact
A	LYS67	B	ARG12	4.81	van der Waals Contact
A	TRP68	B	ARG12	2.81	Hydrogen Bond
A	TRP68	B	GLU112	1.97	van der Waals Contact
A	TRP68	B	LEU113	4.58	Hydrophobic Interaction
A	TRP68	B	GLY111	4.62	van der Waals Contact
A	TRP68	B	VAL118	3.71	Hydrophobic Interaction
A	TRP68	B	ALA117	3.29	Hydrophobic Interaction
A	TRP68	B	TYR164	2.62	van der Waals Contact
A	TRP68	B	TYR24	4.29	van der Waals Contact
A	TRP68	B	GLU22	4.86	van der Waals Contact
A	TRP68	B	ARG145	4.78	van der Waals Contact
A	GLU69	B	TYR24	3.55	van der Waals Contact
A	ASP70	B	ARG12	4.54	van der Waals Contact

A	ASP70	B	LYS10	4.52	van der Waals Contact
A	PRO71	B	PHE11	3.64	Hydrophobic Interaction
A	PRO71	B	LYS10	4.01	van der Waals Contact
A	PRO71	B	TYR24	3.21	van der Waals Contact
A	PRO71	B	ARG12	4.46	van der Waals Contact
A	ILE72	B	LYS10	3.65	van der Waals Contact
A	ALA74	B	ARG12	2.14	Hydrogen Bond
A	ASN75	B	ARG12	3.19	van der Waals Contact
A	ASN75	B	ASP13	1.64	Hydrogen Bond
A	ASN75	B	PHE11	1.82	van der Waals Contact
A	GLY76	B	ASP13	4.54	van der Waals Contact
A	LYS78	B	GLU112	4.97	van der Waals Contact
A	THR80	B	GLU110	4.28	van der Waals Contact
A	THR82	B	GLU110	4.53	van der Waals Contact
A	GLN84	B	VAL187	3.58	van der Waals Contact
A	GLN84	B	ASP186	4.40	van der Waals Contact
A	VAL114	B	GLY111	3.70	van der Waals Contact
A	VAL114	B	GLU110	4.30	van der Waals Contact
A	ASN116	B	GLU110	4.50	van der Waals Contact
A	ASN116	B	SER109	1.82	Hydrogen Bond
A	ASN116	B	GLY111	4.94	van der Waals Contact
A	ARG118	B	SER109	3.01	van der Waals Contact
A	ARG118	B	GLU190	3.01	Salt Bridge

A	ARG118	B	VAL189	3.40	Hydrogen Bond
A	ARG118	B	SER105	2.06	Hydrogen Bond
A	ARG118	B	ILE108	3.04	Hydrogen Bond
A	ARG118	B	LYS106	3.14	van der Waals Contact
A	ARG118	B	PHE107	4.10	van der Waals Contact
A	ARG118	B	ARG104	4.72	van der Waals Contact
A	ASN119	B	GLU190	4.26	van der Waals Contact
A	ARG120	B	VAL189	4.68	van der Waals Contact
A	GLN121	B	VAL189	3.90	van der Waals Contact
A	GLN121	B	VAL187	3.05	van der Waals Contact
A	LYS123	B	GLU110	2.35	Salt Bridge
A	LYS123	B	SER109	2.49	Hydrogen Bond
A	LYS123	B	PHE107	2.50	Hydrogen Bond
A	LYS123	B	ILE108	4.18	van der Waals Contact
A	LYS123	B	LYS106	3.77	van der Waals Contact
A	LYS123	B	VAL187	4.72	van der Waals Contact
A	LYS123	B	VAL189	4.26	van der Waals Contact
A	SER125	B	GLU110	3.44	van der Waals Contact
A	SER125	B	GLY111	4.59	van der Waals Contact
A	TRP127	B	GLY111	2.58	van der Waals Contact
A	TRP127	B	GLU110	3.98	van der Waals Contact
A	TRP127	B	GLU112	2.87	van der Waals Contact
A	TRP127	B	LEU113	4.60	Hydrophobic Interaction

A	TRP127	B	ARG12	4.29	van der Waals Contact
A	SER132	B	ARG17	3.47	van der Waals Contact

Table S4: Intermolecular contacts at the eIF4E-VPg interface for the BRS Cauamé cultivar. The table lists the specific non-covalent interactions between eIF4E (Chain A) and VPg (Chain B), identified from the most representative complex structure. The analysis includes all eIF4E residues located within a 5 Å radius of VPg. For each interacting pair, the table specifies the residues involved, the minimum distance between them in angstroms (Å), and the categorized interaction type (e.g., Hydrogen Bond, Salt Bridge, Hydrophobic, or van der Waals).

**eIF4E**	**Residue 1**	**VPg**	**Residue 2**	**Minimum Distance (Å)**	**Interaction Type**
A	ASP20	B	SER109	2.27	Hydrogen Bond
A	ASP20	B	LEU113	4.87	van der Waals Contact
A	ASP20	B	ILE108	3.59	van der Waals Contact
A	ASP20	B	LYS106	4.16	van der Waals Contact
A	ASP20	B	SER105	4.82	van der Waals Contact
A	ASP20	B	GLU110	4.65	van der Waals Contact
A	GLU21	B	SER109	4.71	van der Waals Contact
A	TRP22	B	SER109	4.73	van der Waals Contact
A	TRP22	B	GLU110	4.45	van der Waals Contact
A	TRP22	B	GLY111	4.31	van der Waals Contact
A	TRP68	B	GLY111	2.19	Hydrogen Bond
A	TRP68	B	SER109	1.87	van der Waals Contact
A	TRP68	B	GLU110	3.74	van der Waals Contact
A	TRP68	B	LEU113	3.18	Hydrophobic Interaction
A	GLU69	B	GLY111	4.13	van der Waals Contact

A	GLU69	B	GLU112	4.46	van der Waals Contact
A	GLU69	B	LEU113	4.64	van der Waals Contact
A	ASP70	B	GLY111	4.63	van der Waals Contact
A	ALA74	B	GLY111	3.59	van der Waals Contact
A	ALA74	B	GLU110	3.73	van der Waals Contact
A	ASN75	B	GLY111	3.12	van der Waals Contact
A	ASN75	B	GLU112	1.87	Hydrogen Bond
A	ASN75	B	GLU110	4.47	van der Waals Contact
A	LYS78	B	GLU110	3.68	Salt Bridge
A	THR80	B	PHE107	3.11	van der Waals Contact
A	THR80	B	LEU80	4.37	van der Waals Contact
A	THR80	B	THR81	4.66	van der Waals Contact
A	THR82	B	VAL187	2.10	Hydrogen Bond
A	THR82	B	ASP186	3.21	Hydrogen Bond
A	THR82	B	PHE107	3.27	van der Waals Contact
A	THR82	B	GLY188	3.81	van der Waals Contact
A	PHE83	B	ASP186	4.88	van der Waals Contact
A	GLN84	B	ASP186	1.84	Hydrogen Bond
A	GLN84	B	GLY188	4.02	van der Waals Contact
A	GLN84	B	VAL187	4.45	van der Waals Contact
A	GLN84	B	VAL189	3.81	van der Waals Contact
A	GLN84	B	GLU185	4.09	van der Waals Contact
A	VAL114	B	GLU110	4.37	van der Waals Contact

A	ASN116	B	GLU110	3.66	van der Waals Contact
A	ARG118	B	GLU190	3.51	Salt Bridge
A	ARG118	B	SER109	2.88	Hydrogen Bond
A	ARG118	B	PHE107	4.81	van der Waals Contact
A	ARG118	B	GLU110	4.79	van der Waals Contact
A	ASN119	B	GLU190	2.70	Hydrogen Bond
A	ARG120	B	GLU190	1.89	Salt Bridge
A	ARG120	B	ASP186	4.24	van der Waals Contact
A	GLN121	B	GLU190	1.96	Hydrogen Bond
A	GLN121	B	GLY188	2.69	Hydrogen Bond
A	GLN121	B	VAL189	3.23	van der Waals Contact
A	GLN121	B	LYS106	4.96	van der Waals Contact
A	LYS123	B	PHE107	2.50	Hydrogen Bond
A	LYS123	B	GLY188	4.27	van der Waals Contact
A	LYS123	B	GLU110	3.36	Salt Bridge
A	LYS123	B	SER109	3.80	van der Waals Contact
A	LYS123	B	ILE108	4.50	van der Waals Contact
A	SER125	B	PHE107	4.91	van der Waals Contact
A	SER125	B	GLU110	4.50	van der Waals Contact
A	TYR150	B	ASP186	4.92	van der Waals Contact
A	GLU152	B	ASN184	3.67	van der Waals Contact
A	GLU152	B	GLU185	4.32	van der Waals Contact
A	GLU152	B	ASP186	4.44	van der Waals Contact

A	THR153	B	ASN184	3.54	van der Waals Contact
A	THR153	B	PRO181	3.55	van der Waals Contact
A	THR153	B	GLY82	3.73	van der Waals Contact
A	THR153	B	ALA182	4.29	van der Waals Contact
A	THR153	B	THR81	4.77	van der Waals Contact
A	GLY155	B	PRO79	3.31	van der Waals Contact
A	GLY155	B	LEU80	3.40	van der Waals Contact
A	GLY155	B	GLY82	3.87	van der Waals Contact
A	GLY155	B	THR81	3.66	van der Waals Contact
A	PHE156	B	PRO79	2.18	Hydrophobic Interaction
A	PHE156	B	LEU80	3.11	Hydrophobic Interaction
A	PHE156	B	THR81	4.83	van der Waals Contact
A	ILE157	B	LEU80	4.11	Hydrophobic Interaction
A	ILE157	B	PHE107	3.08	Hydrophobic Interaction
A	ILE157	B	ILE108	3.53	Hydrophobic Interaction
A	ILE157	B	GLU110	4.69	van der Waals Contact
A	ILE157	B	SER109	4.96	van der Waals Contact
A	PHE158	B	LEU80	3.37	Hydrophobic Interaction
A	PHE158	B	GLU110	4.96	van der Waals Contact

Table S5: Intermolecular contacts at the eIF4E-VPg interface for the BRS Xiquexique cultivar. The table lists the specific non-covalent interactions between eIF4E (Chain A) and VPg (Chain B), identified from the most representative complex structure. The analysis includes all eIF4E residues located within a 5 Å radius of VPg. For each interacting pair, the table specifies the residues involved, the minimum distance between them in angstroms (Å), and the categorized interaction type (e.g., Hydrogen Bond, Salt Bridge, Hydrophobic, or van der Waals).

eIF4E	Residue 1	VPg	Residue 2	Minimum Distance (Å)	Interaction Type
A	THR80	B	SER115	3.81	van der Waals Contact
A	THR80	B	TYR120	3.90	van der Waals Contact
A	MET81	B	SER115	3.58	van der Waals Contact
A	THR82	B	SER115	1.80	Hydrogen Bond
A	THR82	B	LEU113	3.64	van der Waals Contact
A	THR82	B	SER114	3.79	van der Waals Contact
A	THR82	B	GLN116	4.31	van der Waals Contact
A	GLN84	B	ARG12	3.85	van der Waals Contact
A	ARG118	B	GLU112	3.92	Salt Bridge
A	ARG118	B	LEU113	3.65	van der Waals Contact
A	ARG118	B	GLY111	2.10	Hydrogen Bond
A	ARG118	B	SER109	2.26	Hydrogen Bond
A	ARG118	B	GLU110	4.27	van der Waals Contact
A	ASN119	B	GLU112	2.51	Hydrogen Bond
A	ARG120	B	GLU112	1.93	Salt Bridge
A	ARG120	B	GLU190	2.44	Salt Bridge
A	ARG120	B	GLY111	3.85	van der Waals Contact
A	ARG120	B	VAL189	3.69	van der Waals Contact
A	GLN121	B	GLU112	2.10	Hydrogen Bond
A	GLN121	B	LEU113	4.61	van der Waals Contact
A	GLN121	B	SER114	1.60	Hydrogen Bond

A	GLN121	B	SER115	4.90	van der Waals Contact
A	ASP122	B	GLU112	4.72	van der Waals Contact
A	LYS123	B	LEU113	2.74	Hydrogen Bond
A	LYS123	B	MET119	2.19	van der Waals Contact
A	LYS123	B	SER115	4.27	van der Waals Contact
A	LYS123	B	SER114	4.57	van der Waals Contact
A	LYS142	B	ALA44	3.34	van der Waals Contact
A	LYS142	B	LYS45	4.33	van der Waals Contact
A	LYS145	B	LYS40	3.53	van der Waals Contact
A	LYS145	B	LYS43	3.58	van der Waals Contact
A	LYS145	B	GLU163	4.72	van der Waals Contact
A	GLU146	B	LYS40	3.27	Salt Bridge
A	GLU146	B	ALA44	3.69	van der Waals Contact
A	GLU146	B	LYS41	3.98	Salt Bridge
A	PHE147	B	LYS40	3.32	van der Waals Contact
A	LEU148	B	LYS40	1.92	Hydrogen Bond
A	ASP149	B	LYS40	3.90	Salt Bridge
A	TYR150	B	LYS40	3.72	van der Waals Contact
A	TYR150	B	SER115	3.89	van der Waals Contact
A	TYR150	B	GLN116	4.45	van der Waals Contact
A	ASN151	B	LYS43	2.00	Hydrogen Bond
A	ASN151	B	TYR38	1.82	van der Waals Contact
A	ASN151	B	LYS40	3.41	van der Waals Contact

A	ASN151	B	MET30	3.28	van der Waals Contact
A	ASN151	B	THR39	3.13	van der Waals Contact
A	ASN151	B	ASP27	3.86	van der Waals Contact
A	ASN151	B	GLU163	4.25	van der Waals Contact
A	GLU152	B	LYS43	3.98	Salt Bridge
A	GLU152	B	GLU163	3.77	van der Waals Contact
A	GLU152	B	TYR164	1.69	Hydrogen Bond
A	GLU152	B	GLN116	3.51	van der Waals Contact
A	GLU152	B	ALA117	4.13	van der Waals Contact
A	THR153	B	LYS43	3.84	van der Waals Contact
A	THR153	B	GLU163	2.01	Hydrogen Bond
A	THR153	B	TYR164	3.02	van der Waals Contact
A	THR153	B	GLN116	3.76	van der Waals Contact
A	ILE154	B	GLN116	4.93	van der Waals Contact
A	GLY155	B	SER115	4.16	van der Waals Contact
A	GLY155	B	GLN116	4.27	van der Waals Contact
A	GLY155	B	TYR120	4.16	van der Waals Contact
A	PHE156	B	TYR120	3.15	Hydrogen Bond
A	PHE156	B	GLN116	4.67	van der Waals Contact
A	ILE157	B	TYR120	3.50	van der Waals Contact
A	PHE158	B	TYR120	3.18	van der Waals Contact

Table S6: Intermolecular contacts at the eIF4E-VPg interface for the BRS IT85F-2687 cultivar. The table lists the specific non-covalent interactions between eIF4E (Chain A) and VPg (Chain B), identified from the most representative complex structure. The analysis includes all eIF4E residues located within a 5 Å radius of VPg. For each interacting pair, the table specifies the residues involved, the minimum distance between them in angstroms (Å), and the categorized interaction type (e.g., Hydrogen Bond, Salt Bridge, Hydrophobic, or van der Waals).

eIF4E	Residue 1	VPg	Residue 2	Minimum Distance (Å)	Interaction Type
A	ALA15	B	SER105	4.10	van der Waals Contact
A	ALA15	B	GLU102	3.58	van der Waals Contact
A	ALA15	B	TYR120	4.13	van der Waals Contact
A	LYS16	B	GLU102	2.10	Salt Bridge
A	LYS16	B	TYR120	2.86	van der Waals Contact
A	LYS16	B	ASP98	3.95	Salt Bridge
A	LYS16	B	GLU99	4.34	van der Waals Contact
A	SER17	B	TYR120	2.30	Hydrogen Bond
A	SER17	B	GLU102	2.61	Hydrogen Bond
A	SER17	B	ARG94	4.85	van der Waals Contact
A	SER17	B	ASP98	4.59	van der Waals Contact
A	LYS18	B	ARG94	4.41	van der Waals Contact
A	LYS18	B	TYR120	4.71	van der Waals Contact
A	GLN19	B	TYR120	3.11	van der Waals Contact
A	GLN19	B	ARG94	3.10	van der Waals Contact
A	GLN19	B	SER115	3.15	van der Waals Contact
A	GLN19	B	GLN116	3.22	Hydrogen Bond
A	GLN19	B	ASN121	1.84	Hydrogen Bond

A	GLN19	B	GLN97	2.24	van der Waals Contact
A	GLN19	B	ARG162	3.16	van der Waals Contact
A	GLN19	B	GLU165	1.90	Hydrogen Bond
A	ASP20	B	SER115	3.48	van der Waals Contact
A	ASP20	B	GLN116	4.81	van der Waals Contact
A	ASP20	B	TYR120	4.96	van der Waals Contact
A	GLU21	B	SER115	4.82	van der Waals Contact
A	TRP22	B	SER115	4.51	van der Waals Contact
A	ARG54	B	SER109	2.30	Hydrogen Bond
A	ARG54	B	LEU113	2.82	van der Waals Contact
A	ARG54	B	MET119	3.85	van der Waals Contact
A	ARG54	B	SER105	1.97	Hydrogen Bond
A	ARG54	B	ILE108	2.40	Hydrogen Bond
A	ARG54	B	LYS106	3.99	van der Waals Contact
A	ARG54	B	GLU110	4.68	van der Waals Contact
A	ARG54	B	ARG104	4.71	van der Waals Contact
A	TRP79	B	LYS40	4.18	van der Waals Contact
A	ARG118	B	LEU113	2.43	Hydrogen Bond
A	ARG118	B	MET119	3.19	van der Waals Contact
A	ARG118	B	SER115	2.05	Hydrogen Bond
A	ARG118	B	SER114	4.30	van der Waals Contact
A	ARG118	B	SER109	4.77	van der Waals Contact
A	ARG118	B	GLY111	4.94	van der Waals Contact

A	ASN119	B	LEU113	3.79	van der Waals Contact
A	ASN119	B	SER109	1.85	Hydrogen Bond
A	ASN119	B	GLY111	4.58	van der Waals Contact
A	ASN119	B	GLU110	4.37	van der Waals Contact
A	ARG120	B	GLY111	3.05	van der Waals Contact
A	ARG120	B	SER109	3.56	van der Waals Contact
A	ARG120	B	GLU110	2.64	Salt Bridge
A	GLN121	B	GLY111	3.60	van der Waals Contact
A	GLN121	B	LEU113	4.05	van der Waals Contact
A	GLN121	B	GLU112	1.85	Hydrogen Bond
A	LYS123	B	LEU113	3.03	Hydrogen Bond
A	LYS123	B	SER114	3.37	van der Waals Contact
A	LYS123	B	GLU112	4.44	van der Waals Contact
A	LYS123	B	SER115	4.63	van der Waals Contact
A	ALA131	B	ALA44	3.91	Hydrophobic Interaction
A	ALA131	B	LYS40	3.89	van der Waals Contact
A	SER132	B	LYS40	4.03	van der Waals Contact
A	SER132	B	ALA44	4.52	van der Waals Contact
A	SER132	B	LYS41	4.18	van der Waals Contact
A	ASN133	B	LYS40	1.90	Hydrogen Bond
A	GLU134	B	LYS40	3.94	Salt Bridge
A	GLN137	B	LYS40	3.03	van der Waals Contact
A	MET138	B	LYS40	2.97	van der Waals Contact

A	GLU152	B	TYR24	1.79	Hydrogen Bond
A	GLU152	B	LYS10	4.47	van der Waals Contact
A	THR153	B	TYR24	2.25	Hydrogen Bond
A	THR153	B	LYS10	2.49	van der Waals Contact
A	PHE156	B	LYS40	3.65	van der Waals Contact
A	PHE156	B	LYS43	3.85	van der Waals Contact
A	PHE158	B	ALA44	3.32	Hydrophobic Interaction
A	PHE158	B	LYS43	4.04	van der Waals Contact
A	PHE158	B	LYS40	2.89	van der Waals Contact
A	PHE158	B	THR39	4.70	van der Waals Contact
A	PHE158	B	LYS41	4.39	van der Waals Contact

Table S7: Summary of the most frequent eIF4E residues at the interaction interface with VPg**.** This table compiles and ranks the eIF4E residues from the five cultivars analyzed (data from Tables S2–S6) based on their frequency of interaction. The ranking reflects the total number of intermolecular contacts each eIF4E residue establishes with the VPg protein. The top 10 most frequently interacting residues are shown for each cultivar.

**Cultivar**	**Ranking of the Most Frequent Interface Residues in eIF4E–VPg Interactions**									
	**#1**	**#2**	**#3**	**#4**	**#5**	**#6**	**#7**	**#8**	**#9**	**#10**
Bajão	Ser-26	Pro-73	Lys-69	Glu-67	Ile-74	Asp-110	Gly-109	Arg-13	Tyr-64	Asn-132
Boca Negra	Trp-68	Arg-118	Lys-123	Ser-17	Trp-127	Ala-20	Pro-71	Lys-16	Ala-15	Trp-22
BRS Cauamé	Asp-20	Ile-157	Lys-123	Gln-84	Thr-153	Arg-118	Gly-155	Gln-121	Thr-82	Trp-68
BRS Xiquexique	Asn-151	Glu-152	Arg-118	Gln-121	Thr-82	Thr-153	Lys-123	Arg-120	Gly-155	Glu-146
IT85F-2687	Gln-19	Arg-54	Arg-118	Phe-158	Ser-17	Asn-119	Lys-123	Lys-16	Ala-15	Ser-132

Table S8: Hydrogen bond occupancy (%) at the eIF4E–VPg interface for the Bajão cultivar. The table lists the hydrogen bonds formed between eIF4E (Chain A) and VPg (Chain B) during the MD simulation. Occupancy represents the percentage of simulation time that a specific hydrogen bond was maintained, calculated using a donor-acceptor distance cutoff of 3.5 Å and a donor-hydrogen-acceptor angle cutoff of 120 degrees.

Donor	Acceptor	Occupancy (%)
A:GLY25-O	B:SER115-N	62.34
A:SER26-O	B:SER115-OG	10.48
A:HIS108-O	B:LYS2-N	6.27
A:HIS108-ND1	B:LYS2-N	4.80
A:HIS108-O	B:GLY1-N	3.78
A:SER26-O	B:SER105-OG	2.72
A:SER26-OG	B:SER109-O	2.71
A:PRO68-O	B:SER115-N	2.30
A:GLY25-O	B:SER115-OG	1.86
A:HIS108-ND1	B:GLY1-N	1.35
A:HIS108-ND1	B:ARG4-N	1.19
A:HIS108-O	B:ARG4-N	0.98
A:GLY109-O	B:GLY1-N	0.79
A:SER27-OG	B:SER105-O	0.65
A:GLY25-N	B:LEU113-O	0.61
A:GLY109-O	B:LYS2-N	0.60
A:SER26-OG	B:SER105-O	0.30
A:ASP107-O	B:GLY1-N	0.30
A:HIS108-ND1	B:LYS3-N	0.27
A:PRO73-O	B:ASP26-N	0.21
A:GLY109-O	B:TYR28-OH	0.20
A:SER27-N	B:SER115-OG	0.19
A:GLY25-O	B:SER114-N	0.18
A:GLY109-N	B:LYS2-O	0.17
A:SER134-OG	B:GLN7-NE2	0.16
A:GLN21-NE2	B:GLY111-N	0.13
A:ILE74-N	B:ALA25-O	0.11
A:TRP70-N	B:LEU113-O	0.10

A:ASP110-O	B:GLY1-N	0.10
A:SER26-OG	B:LEU113-O	0.06
A:TYR64-OH	B:LYS40-O	0.05
A:SER26-OG	B:ILE108-O	0.04
A:PRO68-O	B:SER114-OG	0.03
A:SER26-N	B:SER115-OG	0.03
A:TRP70-O	B:TYR24-OH	0.02
A:ALA133-N	B:ILE6-O	0.02
A:ASN4-ND2	B:TYR28-OH	0.02
A:SER26-O	B:SER115-N	0.02
A:GLY109-O	B:MET5-N	0.02
A:GLU67-O	B:SER115-OG	0.01
A:SER14-OG	B:VAL189-O	0.01
A:ARG13-NH2	B:SER109-OG	0.01
A:ARG13-NH1	B:SER109-OG	0.01
A:ASN132-ND2	B:GLY1-N	0.01
A:ASN132-ND2	B:LYS2-N	0.01
A:ASP110-N	B:GLY1-O	0.01
A:LYS69-NZ	B:SER114-OG	0.01
A:GLY109-O	B:LYS3-N	0.01

Table S9: Hydrogen bond occupancy (%) at the eIF4E–VPg interface for the Boca Negra cultivar. The table lists the hydrogen bonds formed between eIF4E (Chain A) and VPg (Chain B) during the MD simulation. Occupancy represents the percentage of simulation time that a specific hydrogen bond was maintained, calculated using a donor-acceptor distance cutoff of 3.5 Å and a donor-hydrogen-acceptor angle cutoff of 120 degrees.

Donor	Acceptor	Occupancy (%)
A:ARG120-N	B:GLY188-O	51.55
A:ALA20-O	B:SER115-N	11.72
A:ARG120-N	B:VAL189-O	10.26
A:SER17-O	B:SER115-OG	9.96
A:LYS18-O	B:SER115-OG	8.97
A:SER125-OG	B:GLU110-O	5.88
A:TRP22-N	B:LEU113-O	5.20

A:GLN121-N	B:GLY188-O	4.58
A:LYS16-O	B:SER105-OG	4.27
A:ALA15-O	B:SER105-OG	3.51
A:ASN116-ND2	B:SER109-OG	2.70
A:ASN75-O	B:ASP13-N	1.43
A:ARG118-O	B:GLU190-N	1.12
A:SER17-O	B:TYR120-N	1.02
A:ASN119-ND2	B:GLU190-N	0.94
A:LYS16-O	B:SER115-OG	0.91
A:TRP68-NE1	B:SER114-OG	0.54
A:ASN75-ND2	B:ASP13-N	0.52
A:GLU69-O	B:PHE11-N	0.47
A:GLU21-N	B:LEU113-O	0.38
A:GLN121-NE2	B:GLY188-N	0.37
A:LYS16-O	B:TYR120-OH	0.31
A:SER17-OG	B:ASP98-O	0.26
A:ALA20-O	B:SER115-OG	0.19
A:GLU69-O	B:TYR24-OH	0.19
A:GLN121-N	B:VAL189-O	0.18
A:SER17-N	B:SER105-OG	0.15
A:SER17-OG	B:GLY101-O	0.08
A:ALA15-O	B:TYR120-OH	0.07
A:ARG118-NH1	B:SER109-OG	0.07
A:SER17-O	B:SER105-OG	0.06
A:GLN121-NE2	B:VAL189-N	0.06
A:ASN116-ND2	B:GLY111-N	0.05
A:GLU21-O	B:LEU113-N	0.04
A:SER17-O	B:GLN116-N	0.04
A:LYS18-O	B:TYR120-OH	0.02
A:GLN19-NE2	B:SER115-OG	0.02
A:LYS16-N	B:SER105-OG	0.02
A:ARG120-NH2	B:VAL187-N	0.02
A:ASN116-ND2	B:GLU110-N	0.02
A:ARG118-NH2	B:SER109-OG	0.02

A:ARG120-NH1	B:VAL187-N	0.02
A:SER17-OG	B:MET119-O	0.02
A:GLY76-N	B:ARG12-NH2	0.02
A:SER17-O	B:TYR120-OH	0.01
A:SER17-N	B:TYR120-OH	0.01
A:ARG120-NH1	B:GLY188-N	0.01
A:LYS18-O	B:GLN116-N	0.01
A:TRP68-NE1	B:LEU113-N	0.01
A:GLN19-N	B:SER115-OG	0.01
A:GLN19-O	B:SER115-OG	0.01
A:SER17-N	B:GLY101-O	0.01
A:ARG120-NH2	B:GLY188-N	0.01
A:ARG118-NH2	B:SER109-N	0.01
A:ASN119-O	B:GLU190-N	0.01
A:SER132-N	B:ARG17-NH2	0.01
A:SER132-OG	B:ARG17-NH1	0.01
A:GLN121-N	B:VAL187-O	0.01
A:GLY76-N	B:ARG12-NH1	0.01
A:THR80-OG1	B:GLU110-O	0.01
A:TRP22-NE1	B:GLY111-N	0.01
A:SER17-OG	B:GLU102-N	0.01

Table S10: Hydrogen bond occupancy (%) at the eIF4E–VPg interface for the BRS Cauamé cultivar. The table lists the hydrogen bonds formed between eIF4E (Chain A) and VPg (Chain B) during the MD simulation. Occupancy represents the percentage of simulation time that a specific hydrogen bond was maintained, calculated using a donor-acceptor distance cutoff of 3.5 Å and a donor-hydrogen-acceptor angle cutoff of 120 degrees.

Donor	Acceptor	Occupancy (%)
A:TRP68-O	B:GLY111-N	97.36
A:PHE156-N	B:LEU80-O	46.55
A:PHE156-N	B:PRO79-O	11.56
A:GLN19-O	B:SER109-OG	11.33
A:THR153-OG1	B:GLY82-O	9.86
A:THR82-OG1	B:ASP186-O	7.66
A:THR82-OG1	B:VAL187-O	6.02

A:ASP70-N	B:GLY111-O	4.90
A:THR153-OG1	B:PRO183-O	4.59
A:ASP20-O	B:SER109-OG	3.20
A:THR82-O	B:GLY188-N	2.58
A:THR153-OG1	B:ALA182-O	2.15
A:THR153-OG1	B:ASN184-ND2	2.06
A:THR153-N	B:ALA182-O	0.86
A:PHE158-N	B:LEU80-O	0.74
A:THR153-N	B:PRO183-O	0.52
A:GLN121-NE2	B:GLU190-N	0.51
A:GLN84-NE2	B:VAL189-N	0.45
A:ASN75-ND2	B:GLU112-N	0.29
A:TRP22-N	B:SER109-O	0.27
A:THR153-N	B:ASN184-ND2	0.25
A:GLN121-NE2	B:VAL189-N	0.13
A:TRP68-O	B:GLU112-N	0.11
A:GLN84-NE2	B:VAL187-N	0.09
A:GLU21-N	B:SER109-O	0.09
A:TYR150-OH	B:ASN184-ND2	0.07
A:THR82-OG1	B:GLY188-O	0.07
A:GLN121-O	B:GLU190-N	0.05
A:PHE156-O	B:THR81-OG1	0.05
A:TYR150-OH	B:ASP186-O	0.04
A:THR153-OG1	B:ASN184-N	0.03
A:GLN19-NE2	B:SER109-OG	0.03
A:GLY155-N	B:THR81-O	0.03
A:THR82-OG1	B:GLY188-N	0.02
A:TRP22-N	B:SER109-OG	0.02
A:GLY155-N	B:ASN184-ND2	0.01
A:ARG118-NH2	B:SER109-OG	0.01
A:THR153-OG1	B:PRO79-O	0.01
A:ARG120-NH2	B:VAL189-N	0.01

A:ASP70-O	B:GLU112-N	0.01
A:GLN19-N	B:SER109-OG	0.01
A:ARG118-NH2	B:GLU190-N	0.01
A:ASP20-N	B:SER109-OG	0.01
A:ALA74-O	B:GLY111-N	0.01
A:GLN19-NE2	B:GLU110-N	0.01
A:GLN84-NE2	B:GLY188-N	0.01
A:THR80-OG1	B:THR81-O	0.01
A:GLN121-N	B:GLY188-O	0.01

Table S11: Hydrogen bond occupancy (%) at the eIF4E–VPg interface for the BRS Xiquexique cultivar. The table lists the hydrogen bonds formed between eIF4E (Chain A) and VPg (Chain B) during the MD simulation. Occupancy represents the percentage of simulation time that a specific hydrogen bond was maintained, calculated using a donor-acceptor distance cutoff of 3.5 Å and a donor-hydrogen-acceptor angle cutoff of 120 degrees.

Donor	Acceptor	Occupancy (%)
A:LYS18-O	B:GLY111-N	4.60
A:THR80-O	B:SER115-OG	3.68
A:THR82-N	B:SER115-OG	2.03
A:ALA20-N	B:SER109-O	1.91
A:PHE156-O	B:TYR120-OH	0.46
A:ASN151-O	B:THR147-OG1	0.44
A:GLN121-NE2	B:SER114-OG	0.39
A:ASN151-ND2	B:THR147-OG1	0.38
A:PHE158-N	B:SER105-OG	0.35
A:ASN151-ND2	B:ALA25-N	0.33
A:THR153-O	B:SER115-OG	0.26
A:ASN151-ND2	B:LYS40-N	0.23
A:THR153-OG1	B:GLN116-NE2	0.22
A:GLN19-O	B:SER109-OG	0.22
A:SER139-OG	B:ALA44-O	0.16
A:ALA20-N	B:LYS106-O	0.15
A:GLY155-N	B:GLN116-NE2	0.14

A:GLN19-O	B:GLY111-N	0.13
A:GLN19-NE2	B:SER109-OG	0.09
A:PHE158-N	B:TYR120-OH	0.07
A:THR82-OG1	B:LEU113-O	0.05
A:GLY155-N	B:SER115-OG	0.05
A:SER17-O	B:GLY111-N	0.04
A:ASN151-ND2	B:TYR24-OH	0.04
A:ARG118-NH1	B:GLY111-N	0.04
A:THR82-OG1	B:SER115-N	0.03
A:MET81-N	B:SER115-OG	0.03
A:ARG118-NH2	B:GLY111-N	0.02
A:GLN121-NE2	B:SER114-N	0.02
A:ASN151-O	B:TYR24-OH	0.01
A:THR153-O	B:GLN116-N	0.01
A:THR153-O	B:TYR164-OH	0.01
A:ASN119-N	B:GLY111-O	0.01

Table S12: Hydrogen bond occupancy (%) at the eIF4E–VPg interface for the IT85F-2687 cultivar. The table lists the hydrogen bonds formed between eIF4E (Chain A) and VPg (Chain B) during the MD simulation. Occupancy represents the percentage of simulation time that a specific hydrogen bond was maintained, calculated using a donor-acceptor distance cutoff of 3.5 Å and a donor-hydrogen-acceptor angle cutoff of 120 degrees.

Donor	Acceptor	Occupancy (%)
A:ARG120-N	B:GLY111-O	46.58
A:SER17-O	B:TYR120-OH	3.54
A:SER132-OG	B:LYS40-O	1.77
A:THR153-OG1	B:TYR24-O	1.42
A:THR153-OG1	B:ALA25-O	1.00
A:ARG118-O	B:LEU113-N	0.60
A:LYS18-O	B:TYR120-OH	0.59
A:ASN119-ND2	B:GLY111-N	0.47
A:GLN121-N	B:GLY111-O	0.37
A:GLN19-O	B:SER115-OG	0.27
A:ARG54-N	B:SER109-O	0.25

A:ASN119-ND2	B:SER109-OG	0.13
A:THR153-OG1	B:ALA25-N	0.12
A:SER132-OG	B:LYS41-N	0.12
A:THR153-N	B:TYR24-O	0.10
A:ARG118-NH1	B:SER115-N	0.09
A:GLN121-NE2	B:SER114-OG	0.06
A:THR153-O	B:TYR24-OH	0.06
A:SER132-OG	B:ALA44-O	0.05
A:SER17-O	B:GLU102-N	0.05
A:SER17-O	B:SER105-OG	0.04
A:ARG13-NH1	B:TYR120-OH	0.03
A:ARG118-NH1	B:SER115-OG	0.03
A:GLN19-O	B:TYR120-OH	0.03
A:GLN19-NE2	B:ASP98-N	0.02
A:THR82-OG1	B:ARG12-NH2	0.02
A:MET138-O	B:TYR28-OH	0.02
A:ARG118-NH2	B:SER115-N	0.02
A:ARG54-NH2	B:SER105-OG	0.02
A:GLN19-O	B:GLN116-N	0.01
A:SER17-OG	B:GLY101-O	0.01
A:THR153-OG1	B:ARG12-NH2	0.01
A:THR82-OG1	B:ARG12-NH1	0.01
A:ARG13-NH2	B:TYR120-OH	0.01
A:THR153-OG1	B:ASP26-N	0.01
A:ARG118-NH2	B:TYR120-OH	0.01
A:LYS18-N	B:TYR120-OH	0.01
A:THR153-N	B:TYR24-OH	0.01
A:SER132-OG	B:LYS41-O	0.01
